# CKD-506: A novel HDAC6-selective inhibitor that exerts therapeutic effects in a rodent model of multiple sclerosis

**DOI:** 10.1038/s41598-021-93232-6

**Published:** 2021-07-14

**Authors:** Daekwon Bae, Ji-Young Lee, Nina Ha, Jinsol Park, Jiyeon Baek, Donghyeon Suh, Hee Seon Lim, Soo Min Ko, Taehee Kim, Da Som Jeong, Woo-chan Son

**Affiliations:** 1 Department of Medical Science, Asan Medical Institute of Convergence Science and Technology, Asan Medical Center, University of Ulsan College of Medicine, Seoul, 05505 Republic of Korea; 2 Department of Pathology, Asan Medical Center, University of Ulsan College of Medicine, Seoul, 05505 Republic of Korea; 3 Department of Pharmacology, CKD Research Institute, CKD Pharmaceutical Co, Yongin, 16995 Republic of Korea

**Keywords:** Biologics, Pharmacology, Autoimmunity, Neuroimmunology

## Abstract

Despite advances in therapeutic strategies for multiple sclerosis (MS), the therapy options remain limited with various adverse effects. Here, the therapeutic potential of CKD-506, a novel HDAC6-selective inhibitor, against MS was evaluated in mice with myelin oligodendrocyte glycoprotein_35–55_ (MOG_35–55_)-induced experimental autoimmune encephalitis (EAE) under various treatment regimens. CKD-506 exerted prophylactic and therapeutic effects by regulating peripheral immune responses and maintaining blood–brain barrier (BBB) integrity. In MOG_35–55_-re-stimulated splenocytes, CKD-506 decreased proliferation and downregulated the expression of IFN-γ and IL-17A. CKD-506 downregulated the levels of pro-inflammatory cytokines in the blood of EAE mice. Additionally, CKD-506 decreased the leakage of intravenously administered Evans blue into the spinal cord; CD4^+^ T cells and CD4^−^CD11b^+^CD45^+^ macrophage/microglia in the spinal cord was also decreased. Moreover, CKD-506 exhibited therapeutic efficacy against MS, even when drug administration was discontinued from day 15 post-EAE induction. Disease exacerbation was not observed when fingolimod was changed to CKD-506 from day 15 post-EAE induction. CKD-506 alleviated depression-like behavior at the pre-symptomatic stage of EAE. In conclusion, CKD-506 exerts therapeutic effects by regulating T cell- and macrophage-mediated peripheral immune responses and strengthening BBB integrity. Our results suggest that CKD-506 is a potential therapeutic agent for MS.

## Introduction

Multiple sclerosis (MS), an autoimmune neurological disease, affects 2.5 million individuals worldwide and is characterized by inflammation, demyelination, and axonal damage^1^. Although various therapies have been developed for MS^2^, neurobehavioral dysfunctions and poor quality of life in patients with MS have not been alleviated. After initial diagnosis, approximately 50% of the patients experience relapse within 2 years and 25% develop secondary progressive MS within 20 years^3,4^. The relapse rate decreases by less than 30% after first-line treatment^5^. Meanwhile, second-line treatment is associated with severe side effects, which limit the coverage of patients and underscore the need for periodic monitoring. Natalizumab is not recommended for some patients with MS, while for those undergoing natalizumab therapy, the risk of developing progressive multifocal leukoencephalopathy in the sub-group of patients exhibiting high levels of anti-JC virus antibody is higher than that for patients lacking the anti-JC virus antibody^6^. Patients undergoing fingolimod therapy are monitored with electrocardiograms as they are at an increased risk of presenting bradycardia. Additionally, the high cost of antibody therapy is a major financial burden^7^. Thus, there is a need to develop an efficacious and cost-effective treatment for MS.

Histone deacetylase (HDAC) regulates gene transcription through histone deacetylation^8^. HDAC-mediated epigenetic modulations mediate the pathogenesis of MS^9^. Various studies have demonstrated the role of HDAC in MS using inhibitors, such as sodium phenylbutyrate and trichostatin A. HDAC inhibitors alleviate neurological impairments, inflammation, and demyelination in the CNS of MS animals^9,10^. However, pan-HDAC inhibitors are not available for clinical application due to adverse side effects^11^. Thus, HDAC6 inhibitors have been examined to overcome the adverse effects associated with nonselective HDAC inhibitors. In contrast to other HDACs, HDAC6 is localized in the cytosol and its inhibitors are not associated with adverse events^12^. Furthermore, HDAC6 inhibitors can modulate immune responses and exert neuroprotective effects by promoting the acetylation of various target proteins, such as α-tubulin, cortactin, and Foxp3^13,14^. HDAC6 inhibitors alleviate the symptoms of rheumatoid arthritis (RA) in rodents by downregulating the expression of pro-inflammatory mediators^15,16^, and alleviate symptoms and short-term memory loss in mouse MS models^17^.

Previous studies have demonstrated that CKD-506, a selective HDAC6 inhibitor, modulates the levels of pro-inflammatory cytokines under inflammatory conditions^18^. Additionally, CKD-506 promotes the recovery of renal outcomes, intestinal damage, and arthritis severity in murine systemic lupus erythematosus (SLE), colitis, and RA models, respectively^18–20^. Compared with the initially developed HDAC6 inhibitors (such as tubacin), CKD-506 is a favorable drug candidate^14^. Tubacin was not suitable for drug development owing to its high lipophilicity, rapid metabolization in vivo, and tedious synthesis.

Although HDAC6 has been reported as a potential therapeutic target for autoimmune and neurodegenerative diseases, only one study has reported the therapeutic potential of HDAC6 inhibitors against MS^17^. However, the authors of the study did not elucidate the mechanism of action of the HDAC6 inhibitors against MS. In this study, the effects of CKD-506 on the peripheral and central immune responses were examined in the myelin oligodendrocyte glycoprotein_35–55_ (MOG_35–55_)-induced experimental autoimmune encephalitis (EAE) mouse model. Additionally, the efficacy of CKD-506 against MS was examined under various treatment regimens, such as preventive, therapeutic, drug discontinued, and drug change regimens. Finally, the anti-depressive effects of CKD-506 were also examined.

## Results

### CKD-506 alleviated the clinical symptoms of EAE in mice subjected to the prophylactic regimen

**Figure 1:**
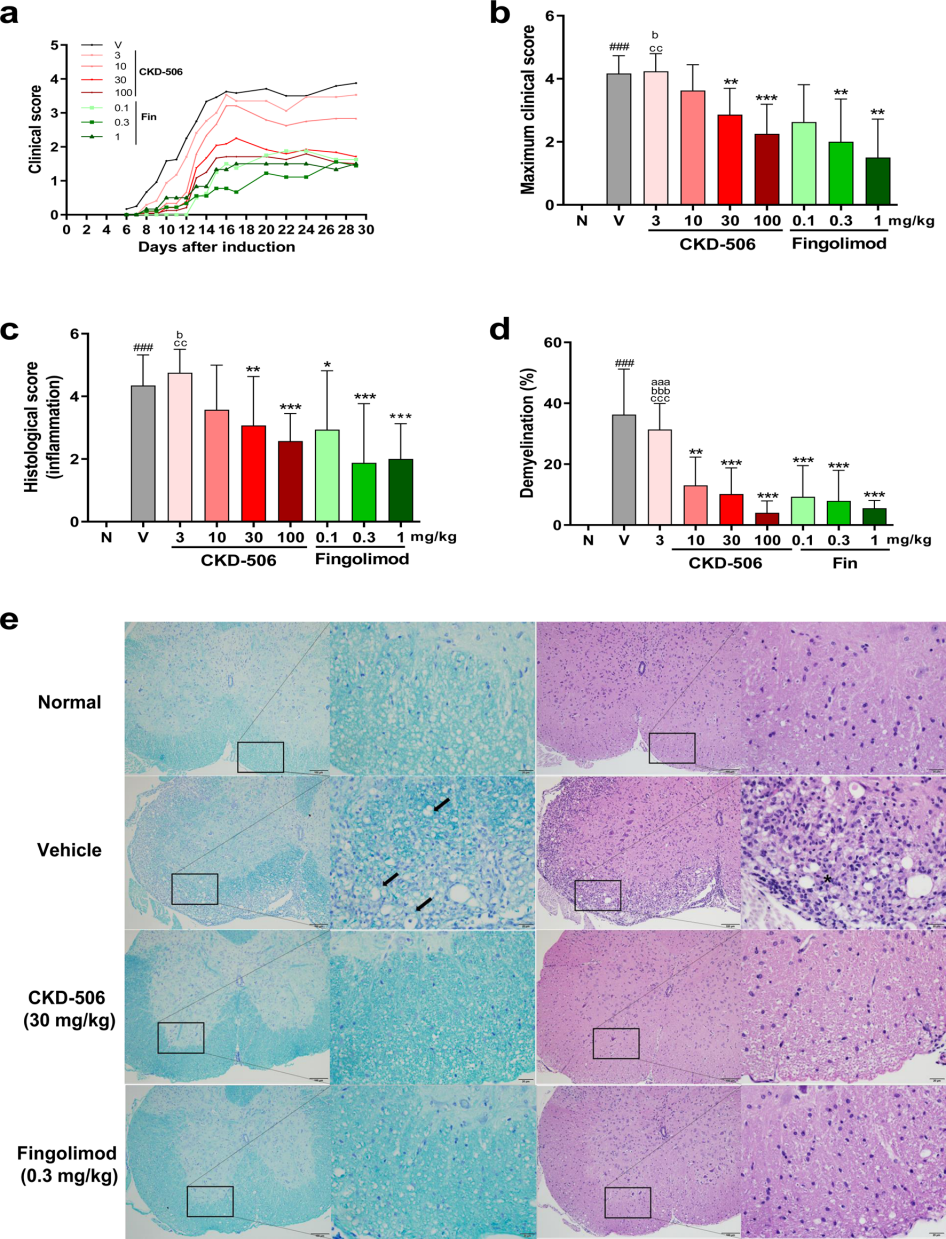
CKD-506 dose-dependently decreased the clinical scores, inflammatory cell infiltration, and demyelination in the experimental autoimmune encephalitis (EAE) mouse model under the prophylactic regimen. C57BL/6 mice were orally administered CKD-506 (3, 10, 30, or 100 mg/kg bodyweight) or fingolimod (0.1, 0.3, or 1 mg/kg bodyweight) daily from day 6 post-myelin oligodendrocyte glycoprotein_35–55_ immunization (**a**–**e**). The clinical score (**a**), maximum clinical score (n = 6–24 per group) (**b**), inflammatory cell infiltration [n = 6 per group for the fingolimod (1 mg/kg)-treated group; n = 8 per group for the fingolimod (0.1 mg/kg)-treated group; n = 9 per group for the fingolimod (0.3 mg/kg)-treated group; n = 17 per group for the non-immunized groups and CKD-506 (3 mg/kg)-treated group; n = 24 per group for the other groups] (**c**), and demyelination [n = 6 per group for the fingolimod (1 mg/kg)-treated group; n = 8 per group for the non-immunized groups, CKD-506 (3 or 10 mg/kg)-treated group, and fingolimod (0.1 or 0.3 mg/kg)-treated group; n = 9 per group for the other groups] (**d**) were examined. (**e**) Representative images of H&E-stained and Luxol fast blue-stained sections. Data are presented as mean ± SD; one-way ANOVA, followed by multiple comparisons post-hoc test with Tukey’s test for (**b**, **d**); Kruskal–Wallis test, followed by multiple comparisons post-hoc test with Dunnett’s test for (**c**). ###*p* < 0.001, non-immunized groups versus vehicle group; **p < 0.05 and ***p < 0.001, drug-treated group versus vehicle group; aaap < 0.001, CKD-506-treated group versus fingolimod (0.1 mg/kg)-treated group; bp < 0.05, bbbp < 0.001, CKD-506-treated group versus fingolimod (0.3 mg/kg)-treated group; ccp < 0.01, cccp < 0.001, CKD-506-treated group versus fingolimod (1 mg/kg)-treated group. N, non-immunized groups; V, vehicle group; Fin, fingolimod-treated group.

To investigate the therapeutic efficacy and effective dose of CKD-506 for MS, the efficacy of CKD-506 was compared with that of fingolimod in MOG_35–55_-induced EAE under the prophylactic regimen. Previous studies have demonstrated that CKD-506 is effective at doses in the range of 10–30 mg/kg bodyweight in rodent models of inflammatory bowel disease, SLE, and RA. Hence, in this study, mice were treated with CKD-506 at a dose range of 3–100 mg/kg bodyweight to examine the effective dose for the prophylactic regimen. Additionally, the dose of fingolimod was determined based on the blood exposure level in humans. The mouse equivalent dose of fingolimod is 0.1–0.3 mg/kg bodyweight^5^. CKD-506 dose-dependently decreased the clinical scores and delayed the disease onset time. The clinical scores were significantly lower, while the disease onset was significantly delayed in the groups treated with CKD-506 at doses of ≥ 30 mg/kg bodyweight than in the vehicle-treated group [Fig. 1a, Table 1; F(8, 144) = 62.65; *p* < 0.001; one-way Analysis of variance (ANOVA)]. Furthermore, the mean clinical score and final clinical score in the CKD-506 (30 mg/kg bodyweight)-treated group did not differ from that in the fingolimod (1 mg/kg bodyweight)-treated group [Fig. 1b; F(8, 144) = 45.10; *p* < 0.001; one-way ANOVA, and Table 1; F(8, 144) = 62.65; *p* < 0.001; one-way ANOVA].

**Table 1:** Values of the mean onset time, mean clinical score, maximum clinical score, and final clinical score in the CKD-506- and fingolimod-treated experimental autoimmune encephalitis (EAE) groups.

Values for clinical parameters (mean ± SD)				
Treatment	Mean onset time	Mean clinical score*	Maximum clinical score	Final clinical score
Vehicle (n = 24)	9.3 ± 2.2	2.5 ± 0.7	4.2 ± 0.6	3.9 ± 0.9
CKD-506 (3 mg/kg bodyweight; n = 17)	10.6 ± 2.3	2.2 ± 0.7 aa, bbb	4.2 ± 0.6 b, cc	3.5 ± 1.2 b
CKD-506 (10 mg/kg bodyweight; n = 24)	12.1 ± 2.3	1.7 ± 0.5 b	3.6 ± 0.6	2.8 ± 0.8
CKD-506 (30 mg/kg bodyweight; n = 24)	12.9 ± 1.7**	1.1 ± 0.4***	2.9 ± 0.9**	1.7 ± 0.8***
CKD-506 (100, n = 24)	13.3 ± 1.8***	0.9 ± 0.5***	2.3 ± 0.9***	1.5 ± 0.9***
Fin * (0.1 mg/kg bodyweight; n = 8)	15.1 ± 2.9**	0.8 ± 0.4***	2.6 ± 1.2	1.6 ± 1.3*
Fin (0.3 mg/kg bodyweight; n = 9)	15.1 ± 4.4**	0.6 ± 0.4***	2.1 ± 1.5**	1.4 ± 1.8**
Fin (1 mg/kg bodyweight; n = 6)	14.2 ± 7.7**	0.8 ± 0.6**	1.5 ± 1.2**	1.5 ± 1.2*

Data are presented as mean ± SD (*p < 0.05, **p < 0.01, and ***p < 0.001, drug-treated group versus vehicle group, aa *p* < 0.01, CKD-506-treated group versus fingolimod (0.1 mg/kg)-treated group, bp < 0.05, bbbp < 0.001, CKD-506-treated group versus fingolimod (0.3 mg/kg)-treated group, ccp < 0.01, CKD-506-treated group versus fingolimod (1 mg/kg)-treated group). Results were analyzed using Kruskal–Wallis test, followed by multiple comparisons post-hoc test with Dunnett’s test.

*The mean clinical score was calculated as follows:

The mean clinical score = the accumulated score/evaluated days.

*Fin, Fingolimod-treated group.

### CKD-506 alleviated spinal cord damage in mice with EAE

Histological analysis was performed to examine the therapeutic effects of CKD-506. The spinal cord section was stained with Luxol fast blue (LFB) to examine the demyelination pattern. The LFB-stained area in the vehicle group (36.25 ± 14.19%) was approximately 36% lesser than that in the non-immunized groups (0.07 ± 0.04%). The area with decreased LFB staining intensity was correlated with immune cell infiltration (Fig. 1e). The LFB-stained area in the CKD-506-treated and fingolimod-treated groups was approximately 5–15% lower than that in the vehicle-treated group [Fig. 1d; F(8, 68) = 3.575; *p* = 0.0016; one-way ANOVA].

### CKD-506 decreased the infiltration of T cells and macrophages in the spinal cord of mice with EAE

**Figure 2:**
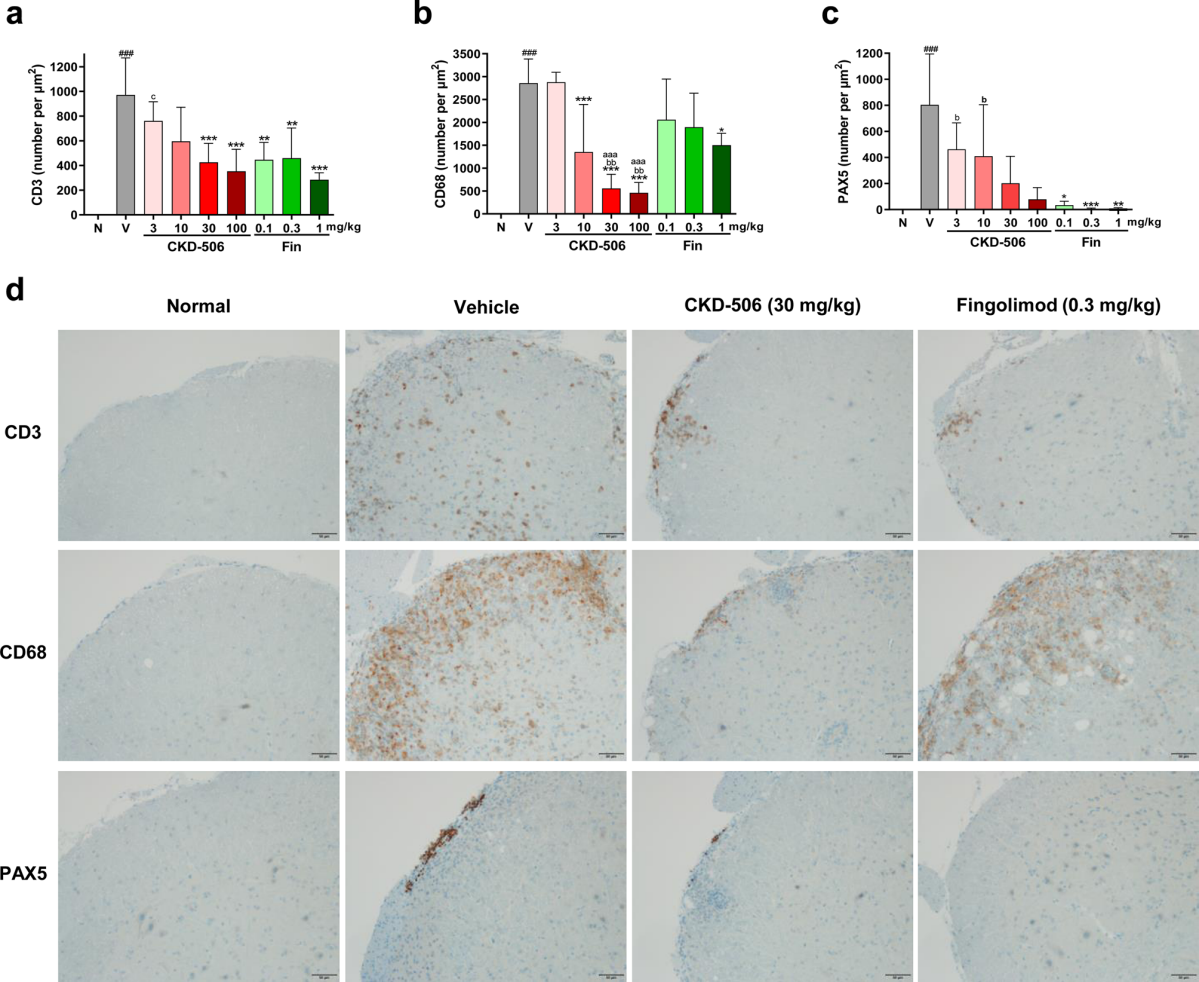
CKD-506 decreased the infiltration of CD3 + T cells and CD68 + microglia/macrophages into the spinal cord parenchymal region in the experimental autoimmune encephalitis (EAE) mouse model on day 29 post-EAE induction. C57BL/6 mice [n = 6 per group for the fingolimod (1 mg/kg)-treated group; n = 7 per group for the non-immunized groups, CKD-506 (3, 10 mg/kg)-treated groups, and fingolimod (0.1 mg/kg)-treated group; n = 8 per group for the other groups] were orally administered CKD-506 (10, 30, or 100 mg/kg) or fingolimod (0.1 or 0.3 mg/kg) daily from day 6 post-myelin oligodendrocyte glycoprotein_35–55_ immunization (**a**–**d**). The number of CD3 + T cells (**a**), CD68 + microglia/macrophages (**b**), and PAX5-positive B cells (**c**) in the spinal cord were analyzed. (**d**) Representative images of immunohistochemical analysis are shown. Data are presented as mean ± SD; one-way ANOVA, followed by multiple comparisons post-hoc test with Tukey’s test for (**a**, **b**); Kruskal–Wallis test, followed by multiple comparisons post-hoc test with Dunnett’s test for (**c**). ###*p* < 0.001, non-immunized groups versus vehicle group; *p < 0.05, **p < 0.01, and ***p < 0.001, drug-treated group versus vehicle group; aaap < 0.001, CKD-506-treated group versus fingolimod (0.1 mg/kg)-treated group; bp < 0.05, bbp < 0.01, CKD-506-treated group versus fingolimod (0.3 mg/kg)-treated group; cp < 0.05, CKD-506-treated group versus fingolimod (1 mg/kg)-treated group. N, non-immunized groups; V, vehicle group; Fin, fingolimod-treated group.

The infiltration of peripheral immune cells, such as T cells and macrophages, into the spinal cord can induce adverse clinical symptoms, including myelin and axonal pathology in EAE. Thus, the number of immune cells in the spinal cord was examined on day 29 post-EAE induction. The H&E-stained spinal cord (L3–L5) sections of the vehicle group exhibited enhanced infiltration of immune cells [Fig. 1c; F(8, 144) = 26.14; *p* < 0.001; one-way ANOVA]. The highest infiltration of immune cells was observed in the peripheral region of white matter (Fig. 1e). CKD-506 decreased the infiltration of immune cells into the spinal cord. The decrease in the infiltration of immune cells in the spinal cord of the CKD-506 (30 mg/kg bodyweight)-treated group was similar to that in the spinal cord of the fingolimod (0.1 mg/kg bodyweight)-treated group (Fig. 1c) [Fig. 1c; F(8, 144) = 26.14; *p* < 0.001; one-way ANOVA]. Immunohistochemical (IHC) analysis revealed that the expression of CD3 (T cell marker) and CD68 (macrophage/microglia marker), which were mainly localized in the parenchyma of white matter, in the vehicle group was higher than that in the non-immunized groups (Fig. 2d). Moreover, the expression of PAX5 (B cell marker) was upregulated in the spinal cord of the vehicle group. However, most PAX5-positive cells were localized in the meninges and pia mater and not the parenchyma, which suggests that B cells are not associated with the clinical symptoms of mice with EAE (Fig. 2d). Treatment with CKD-506 or fingolimod decreased the number of T cells and B cells in the spinal cord. The decrease in the number of T cells in the CKD-506 (30 mg/kg bodyweight)-treated group did not differ from that in the fingolimod-treated group [Fig. 2a; F(8, 57) = 8.844, *p* < 0.001; one-way ANOVA, and 2C; kai square(9) = 46.52, *p* < 0.0001; one-way ANOVA]. Additionally, treatment with CKD-506 (10 mg/kg bodyweight) decreased the number of CD68-positive cells, which was not observed after treatment with fingolimod [Fig. 2b; F(8, 57) = 17.93; *p* < 0.0001; one-way ANOVA]. Flow cytometric analysis showed that CKD-506 (30 mg/kg) reduced the levels of CD4^+^ T cells and CD4^−^CD11^+^CD45^+^ macrophage/microglia in the spinal cord of EAE mice [Supplementary data 1; T(4) = 3.835; *p* = 0.0189 for CD4^+^ T cells and T(4) = 5.086 *p* = 0.0071 for CD4^−^CD11^+^CD45^+^ macrophage/microglia; unpaired *t*-test]. These findings suggest that CKD-506 alleviated the clinical symptoms in mice with EAE by inhibiting the infiltration of T cells and macrophages into the spinal cord. In contrast, fingolimod decreased only T cell infiltration.

### CKD-506 downregulated the levels of Th1 cell-related and macrophage-related pro-inflammatory cytokines in the spinal cord and peripheral blood of mice with EAE

**Figure 3:**
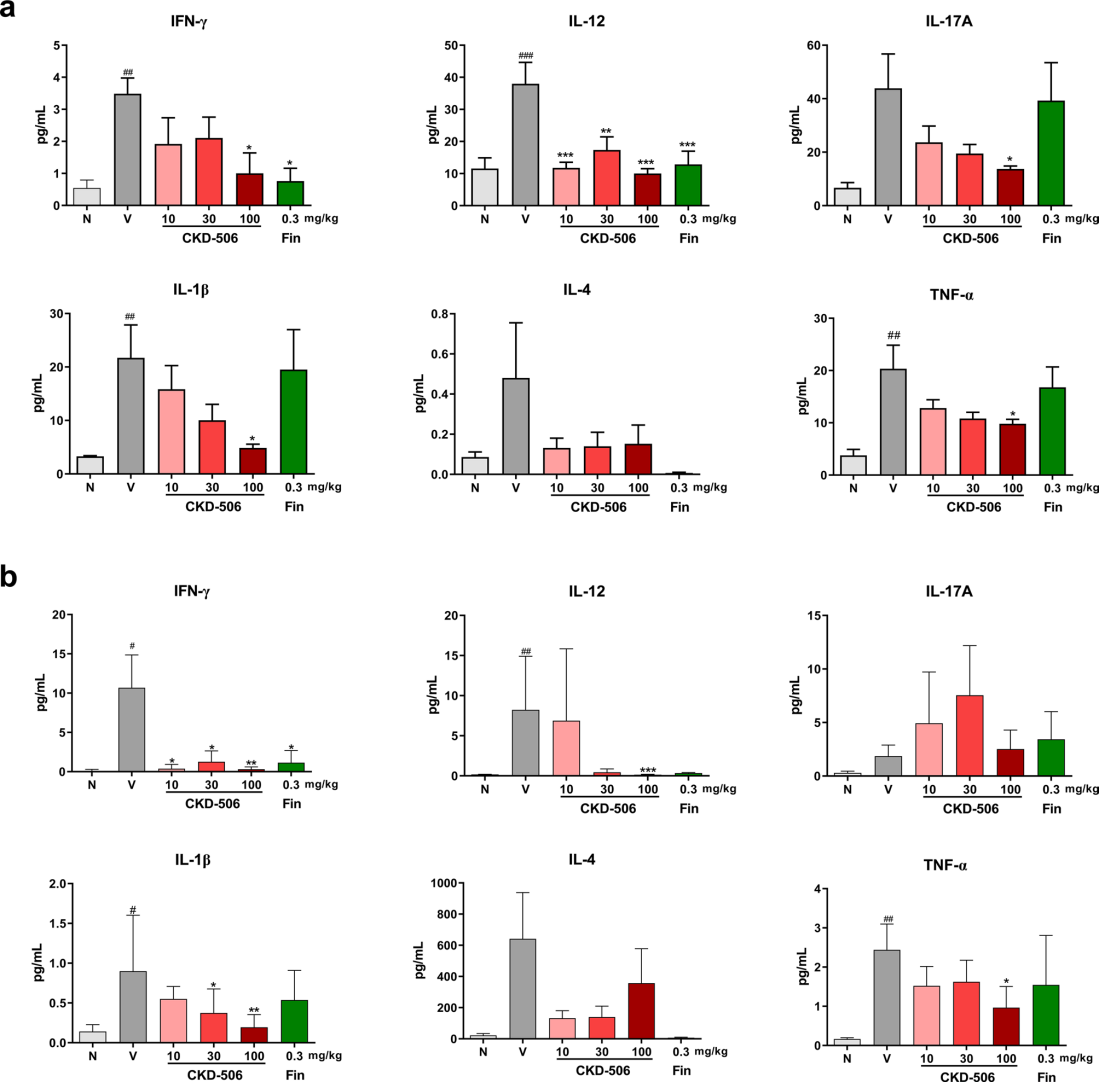
CKD-506 downregulated the levels of Th1/17 and macrophage-secreted pro-inflammatory cytokines in the blood and spinal cord of mice with experimental autoimmune encephalitis (EAE) on day 29 post-EAE induction. C57BL/6 mice (n = 5 per group for the non-immunized groups; n = 7 per group for the other groups) were orally administered CKD-506 (10, 30, or 100 mg/kg) and fingolimod (0.3 mg/kg) daily from day 6 post-myelin oligodendrocyte glycoprotein_35–55_ immunization and pro-inflammatory cytokine levels in their blood or spinal cord were evaluated on day 29 post-induction (**a**, **b**). The levels of IFN-γ, IL-12, IL-17A, IL-1β, IL-4, and TNF-α in the plasma (**a**) and supernatant from spinal cord homogenate (**b**) were quantified using the multiplex cytokine assay. Data are presented as mean ± SD; one-way ANOVA, followed by Dunnett’s post-hoc for IFN-γ, IL-12, IL-1β, TNF-α from (**a**) and IFN-γ, IL-17A, IL-1β, TNF-α from (**b**); Kruskal–Wallis test, followed by post-hoc test with Dunnett’s test for IL-17A, IL-4 from (**a**) and IL-12, IL-4 from (**b**). #*p* < 0.05, ##*p* < 0.01, and ###*p* < 0.001, non-immunized groups versus vehicle group; *p < 0.05, **p < 0.01, and ***p < 0.001, drug-treated group versus vehicle group. N, non-immunized groups; V, vehicle group; Fin, fingolimod-treated group.

Next, the effect of CKD-506 on the immune-mediated inflammatory response was examined. The levels of immune cell-related cytokines in the spinal cord and peripheral blood were analyzed using the multiplex assay. The levels of Th1 cell-related and macrophage-related cytokines, such as IFN-γ, IL-12, TNF-α, and IL-1β were upregulated in the spinal cord and blood of the vehicle group on day 29 post-EAE induction. However, the levels of IL-17, secreted by activated Th17 cells, and IL-4, secreted by the Th2 cells, were not affected in the blood and spinal cord of the vehicle group. Treatment with CKD-506 downregulated the levels of Th1 cell- and macrophage-secreted cytokines in the spinal cord and blood of the vehicle group (Fig. 3). However, treatment with fingolimod decreased the levels of IFN-γ and IL-12 but not those of TNF-α, and IL-1β in the peripheral blood and spinal cord of the vehicle group. These findings suggest that CKD-506 suppressed Th1 cell- and M1 macrophage-mediated immune responses, whereas fingolimod suppressed only T cell-mediated immune responses.

### CKD-506 inhibited the proliferation of peripheral immune cells in the MOG-stimulated splenocytes

**Figure 4:**
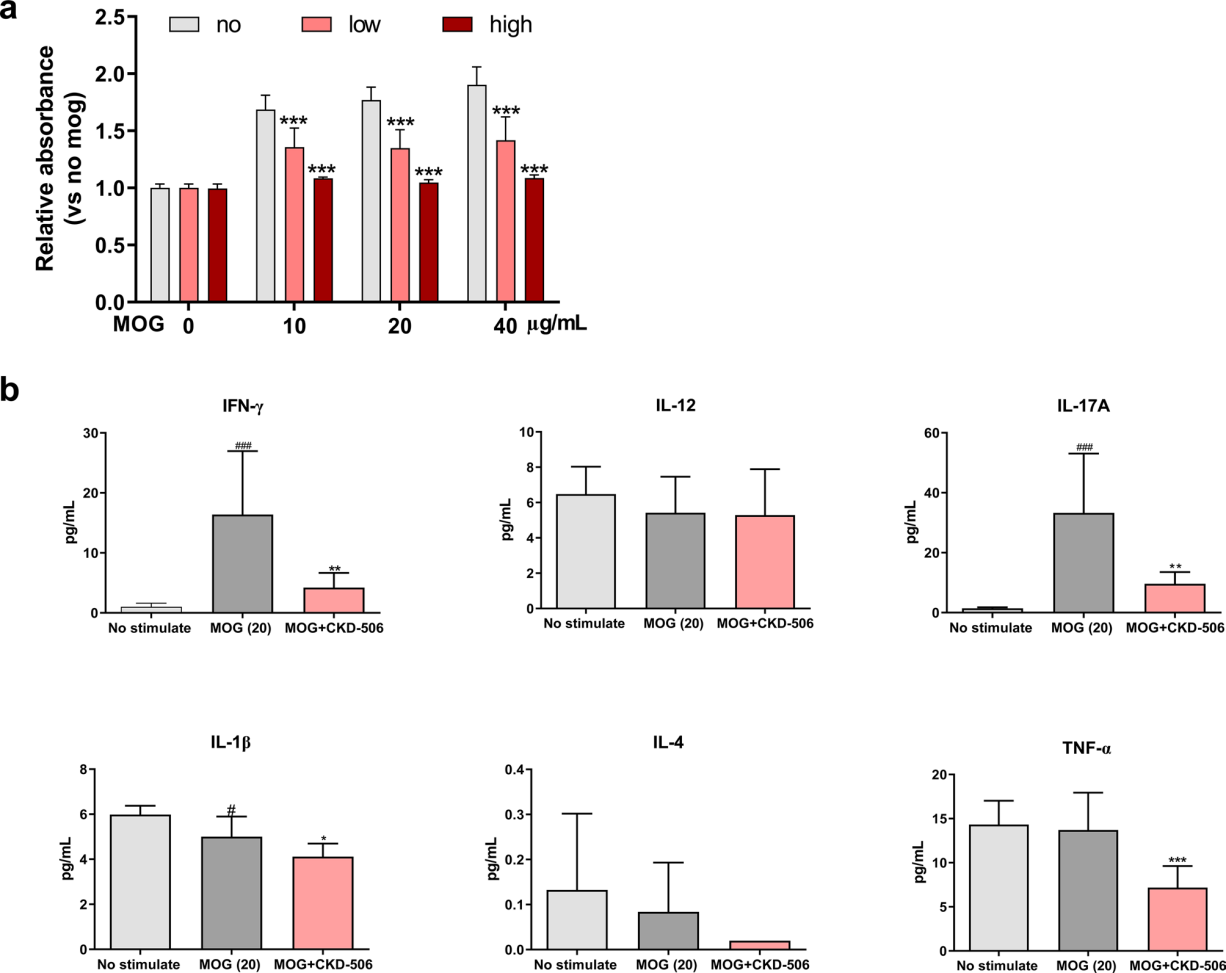
CKD-506 decreased the levels of Th1/17-secreted pro-inflammatory cytokines and decreased the proliferation of myelin oligodendrocyte glycoprotein_35–55_ (MOG_35–55_)-stimulated splenocytes. Mice induced using MOG_35–55_ and pertussis toxin were sacrificed by cervical dislocation on day 12 post-induction and the splenocytes were isolated. Mice were not treated with any drugs, including vehicle. CCK8 solution was used to examine the proliferation of the splenocytes incubated with the MOG_35–55_ peptide (10, 20, and 40 μg/mL), vehicle, and CKD-506 (1 and 3 μM) for 72 h at 37 °C. The absorbance values of the mixtures were analyzed 3 h later. The data were obtained from three independent experiments with three replicates (**a**). Televels of IFN-γ, IL-12, IL-17A, IL-1β, IL-4, and TNF-α in the culture supernatant of the splenocytes stimulated with MOG_35–55_ (20 μg/mL) for 72 h at 37 °C and those in the culture supernatant of stimulated splenocytes co-incubated with CKD-506 (3 μM) were examined (n = 9 per group) (**b**). Data are presented as mean ± SD. Two-way ANOVA, followed by Bonferroni’s post-hoc test for (**a**); one-way ANOVA, followed by Dunnett’s post-hoc (**b**). #*p* < 0.05, and ###*p* < 0.001, non-stimulated groups versus MOG-stimulated group (vehicle group); *p < 0.05, **p < 0.01, and ***p < 0.001, drug-treated group versus vehicle group. No (gray color), vehicle treatment; low (light red), 1 μM CKD-506 treatment; high (dark red), 3 μM CKD-506 3 μM treatment.

The progression of EAE is associated with the activation of peripheral immune responses against MOG, including the differentiation, activation, and expansion of immune cells and the secretion of various pro-inflammatory cytokines in the peripheral lymphoid organs^21,22^. To evaluate the role of suppressed peripheral immune response activation in spinal cord damage, the peripheral autoimmune response against MOG was examined using CCK8 and cytokine multiplex assays and the proportion of CD3^+^ T cells in the blood of EAE mice was assessed. The results of the CCK8 assay revealed that CKD-506 (1 or 3 μM) significantly decreased the proliferation of MOG-stimulated splenocytes [Fig. 4a; F(3, 18) = 28.07; *p* < 0.001; two-way ANOVA]. The expression levels of IFN-γ and IL-17A in the MOG (20 μg/mL)-stimulated splenocytes of the vehicle group were higher than those in the MOG (20 μg/mL)-stimulated splenocytes of the non-immunized groups. However, the expression levels of IL-1β and TNF-α in the MOG-stimulated splenocytes were similar between the EAE and normal mice. Treatment with CKD-506 (3 μM) significantly downregulated the expression levels of IFN-γ, IL-17A, IL-1β, and TNF-α, which are mainly secreted from the Th1/Th17 cells and M1 macrophages (Fig. 4b). Results of the one-way ANOVA of the cytokine levels are shown in supplementary data. CKD-506 treatment (30 mg/kg) reduced the proportion of CD3^+^ and CD3^+^CD8^−^ T cells in the blood of EAE mice at 21 days post-induction [Supplementary data; F(2, 9) = 32.36; *p* < 0.001, F(2, 9) = 33.93; *p* < 0.001; one-way ANOVA].

### CKD-506 enhanced blood–brain barrier (BBB) integrity in mice with EAE

**Figure 5:**
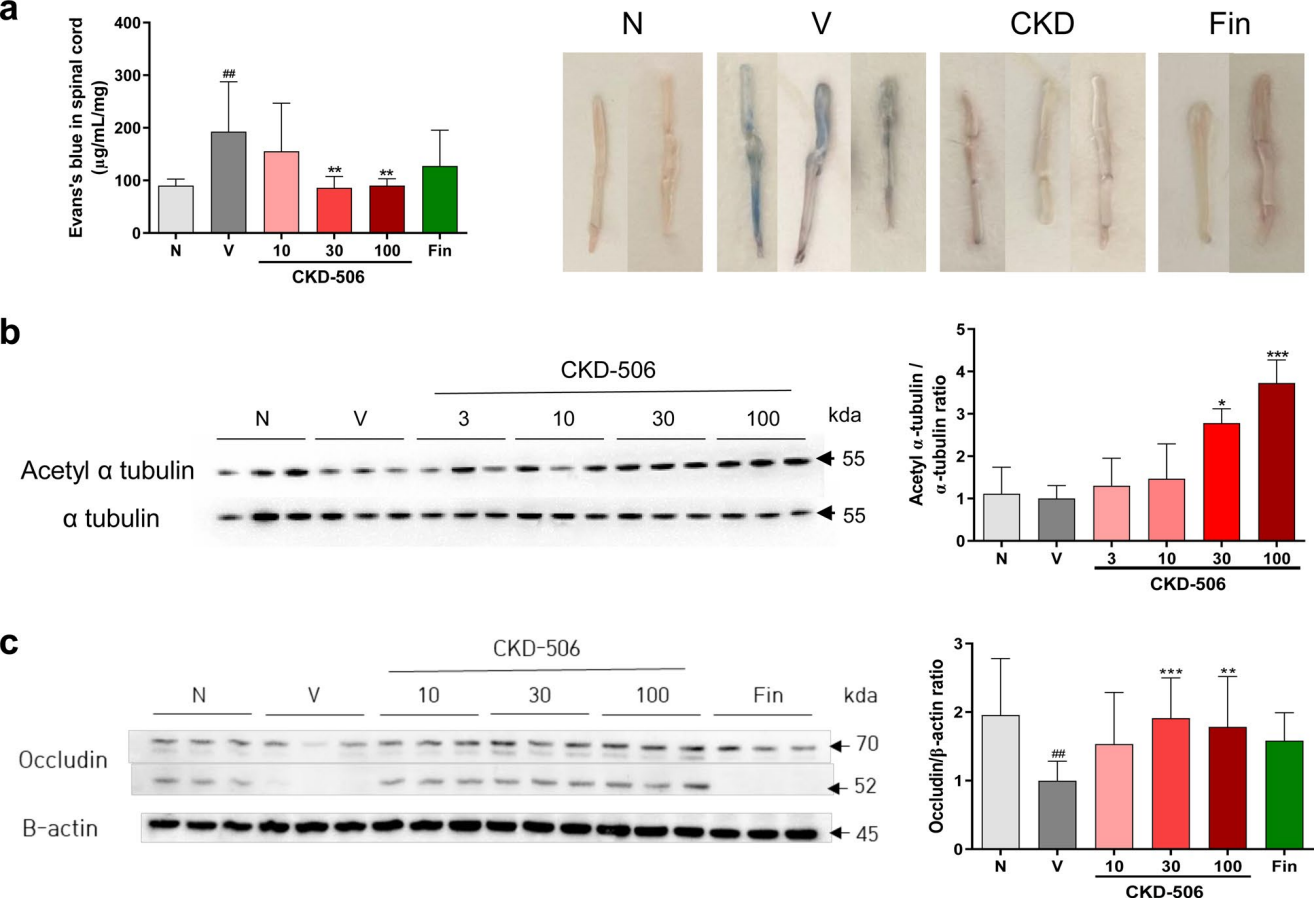
CKD-506 protected blood–brain barrier integrity in mice with experimental autoimmune encephalitis (EAE) by upregulating occludin expression. Mice were treated with CKD-506 (10, 30, or 100 mg/kg bodyweight) and fingolimod (0.3 mg/kg bodyweight) from day 6 post-EAE induction. The volume of Evans blue dye and the levels of acetylated α-tubulin and occludin in the spinal cord were analyzed 15 days later (**a**–**c**). The infiltration of Evans blue dye in the spinal cord of mice with EAE was quantified 1 h after the intravenous injection of the dye (n = 9 per group for the vehicle group; n = 8 per group for the other groups). Additionally, the protein expression levels of acetylated α-tubulin (n = 3 per group) (**b**) and occludin [n = 8 per group for the non-immunized groups and fingolimod (1 mg/kg) treated group, n = 14 per group for the other groups] (**c**) in the spinal cord of mice with EAE were evaluated 1 h after the final drug administration on day 21 post-induction. Data are presented as mean ± SD and analyzed using one-way ANOVA, followed by Dunnett’s post-hoc test. #*p* < 0.05, ##*p* < 0.01, non-immunized groups versus vehicle group; *p < 0.05, **p < 0.01, and ***p < 0.001, drug-treated group versus vehicle group. N, non-immunized groups; V, vehicle group; Fin, fingolimod (0.3 mg/kg bodyweight)-treated group; CKD, CKD-506 (30 mg/kg bodyweight)-treated group.

Translocation of the peripheral immune cells through BBB contributes to the development of MS. Previous studies have demonstrated that the maintenance of BBB integrity can prevent the induction and exacerbation of symptoms in mice with EAE^23^. This study examined the extravasation of the Evans blue (EB) dye and the expression level of occludin, an endothelial tight junction protein, in the spinal cord to determine the effect of CKD-506 and fingolimod on BBB integrity in the vehicle group on day 21 post-EAE induction. Additionally, the levels of acetylated α-tubulin in the spinal cord were also examined to confirm the passage of CKD-506 through the BBB to exert its effect on the CNS. EB leakage was detected in the spinal cord of the vehicle group but not in the non-immunized groups, while the EB volume in the spinal cord of the CKD-506 (30 and 100 mg/kg bodyweight)-treated group was significantly lower than that in the spinal cord of the vehicle-treated group [Fig. 5a; F(5, 43) = 4.122; *p* = 0.0038; one-way ANOVA]. However, the EB volume in the spinal cord did not significantly differ between the fingolimod (0.3 mg/kg bodyweight) and vehicle groups. The expression levels of acetylated α-tubulin and occludin in the CKD-506 (30 or 100 mg/kg bodyweight)-treated group were significantly higher than those in the vehicle group (Fig. 5b; F(5,12) = 10.97; *p* = 0.0004; one-way ANOVA and Fig. 5c; F(5,66) = 4.361; *p* = 0.0017; one-way ANOVA, respectively). These results suggest that CKD-506 (30 mg/kg bodyweight) reached the CNS and enhanced BBB integrity by upregulating occludin expression.

### CKD-506 alleviated clinical symptoms in mice with EAE under the therapeutic regimen

**Figure 6:**
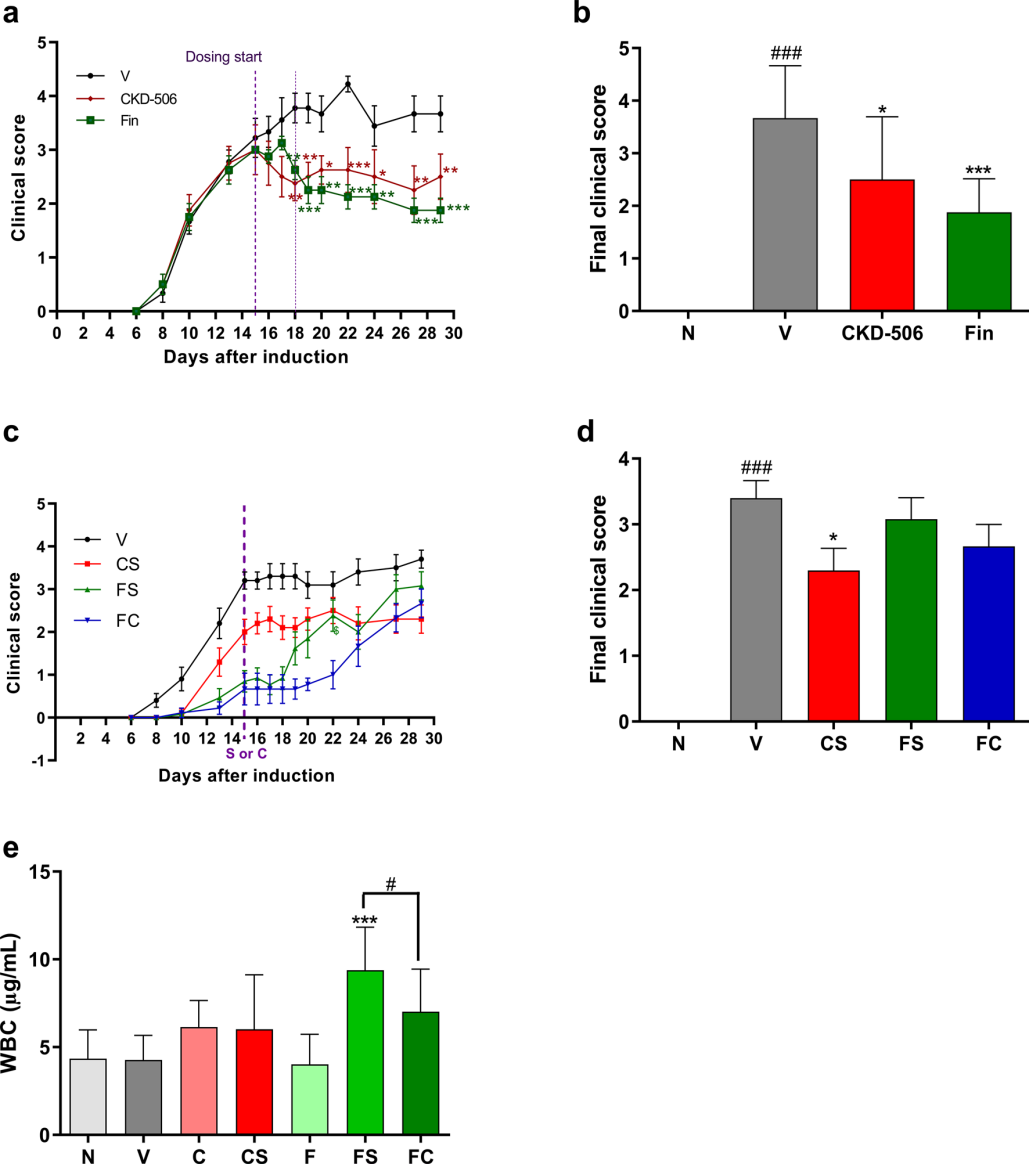
CKD-506 decreased the clinical scores in mice with experimental autoimmune encephalitis (EAE) under the therapeutic regimen. C57BL/6 (n = 9 per group for the non-immunized groups and vehicle group; n = 8 per group for the other groups) mice were orally administered CKD-506 (30 mg/kg bodyweight) and fingolimod (0.3 mg/kg bodyweight) daily from day 15 post-myelin oligodendrocyte glycoprotein_35–55_ immunization (**a**, **b**). The clinical score (**a**) and final clinical score (**b**) were determined. The CKD-506-treated group exhibited alleviated clinical scores even after CKD-506 discontinuation and delayed disease exacerbation after fingolimod discontinuation (**c**, **d**). Mice (n = 12 per group for FS; n = 10 per group for the other groups) were treated with CKD-506 (30 mg/kg bodyweight) and fingolimod (0.3 mg/kg bodyweight) from days 6 to 14 post-induction. Treatment was discontinued or changed from fingolimod to CKD-506 on day 15 post-induction. The clinical score (**c**), final clinical score (**d**), and the number of white blood cells in the blood (**e**) were evaluated. Data are presented as mean ± SD. Two-way ANOVA, followed by Bonferroni post-hoc test for (**a**, **c**); one-way ANOVA, followed by Dunnett’s post-hoc test for (**b**, **d**, **e**). #*p* < 0.05, ###*p* < 0.001, non-immunized groups versus vehicle group; *p < 0.05, **p < 0.01, and ***p < 0.001, drug-treated group versus vehicle group; $*p* < 0.05, FS versus FC. N, non-immunized groups; V, vehicle group; Fin, fingolimod-treated group; CS, CKD-506-treated group in which dosing of CKD-506 was discontinued from day 15 post-induction; FS, fingolimod-treated group in which treatment was discontinued from day 15 post-induction; FC, the group in which fingolimod was replaced with CKD-506 on day 15 post-induction; S, dosing stop; C, treatment change from fingolimod to CKD-506.

To investigate the therapeutic potential of CKD-506 in severe MS, the efficacies of CKD-506 and fingolimod against EAE were comparatively analyzed under the therapeutic regimen. CKD-506 and fingolimod exhibited potent therapeutic efficacies at doses of 30 and 0.3 mg/kg bodyweight, respectively, under the prophylactic regimen. Hence, the same doses of CKD-506 and fingolimod were used for the therapeutic regimen. CKD-506 decreased the clinical scores immediately after treatment. The decrease in clinical scores was significant from day 3 post-treatment [Fig. 6a; F(2,308) = 34.18; *p* < 0.0001, two-way ANOVA, Table 2; F(3,30) = 4.750; *p* = 0.0079, one-way ANOVA]. Moreover, the mean clinical score and final clinical score in the CKD-506-treated group was significant and comparable with that in the fingolimod-treated group [Fig. 6b; F(3, 30) = 4.331; *p* = 0.0119; one-way ANOVA, Table 2; F(3, 20) = 4.750; *p* = 0.0079; one-way ANOVA]. Additional blood cytokine analysis at 29 days after induction showed that CKD-506 (30 mg/kg) reduced the levels of IFN-γ and TNF-α, but not of IL-12, IL-17A, and IL-1β [Supplementary data; chi square(3) = 17.91; *p* = 0.0005; Kruskal–Wallis test for IFN-γ and F(3, 24) = 14.30; *p* < 0.0001; one-way ANOVA for TNF- α].

**Table 2:** Values of the mean clinical score, maximum clinical score, and final clinical score in the CKD-506- and fingolimod-treated experimental autoimmune encephalitis (EAE) groups under the therapeutic regimen.

(A) Values for clinical parameters (mean ± SD) in therapeutic mode			
Treatment	Mean clinical score* (days from 15 to 29)	Maximum clinical score (days from 15 to 29)	Final clinical score
Vehicle (n = 9)	3.6 ± 0.8	4.2 ± 0.4	3.7 ± 1.0
CKD-506 (30 mg/kg; n = 8)	2.6 ± 1.0**	3.4 ± 1.1	2.5 ± 1.2*
Fin * (0.1 mg/kg; n = 8)	2.4 ± 0.4**	3.1 ± 0.4	1.9 ± 0.6***

Data are presented as mean ± SD (*p < 0.05, **p < 0.01 and ***p < 0.001, drug-treated group vs. vehicle group). Results were analyzed using Kruskal–Wallis test, followed by multiple comparisons post-hoc test with Dunnett’s test for maximum clinical score and using one-way ANOVA followed by Dunnett’s post-hoc test for mean clinical score and final clinical score.

*The mean clinical score was calculated as follows:

The mean clinical score = the accumulated score / evaluated days.

*Fin, Fingolimod-treated group.

### CKD-506 exerted therapeutic efficacy after drug discontinuation and delayed the exacerbation of EAE after fingolimod administration was discontinued

Clinically, the discontinuation of fingolimod administration results in the exacerbation of MS symptoms. Thus, this study examined the therapeutic efficacy of CKD-506 after the discontinuation of drug administration, as well as the preventive effects of CKD-506 on the exacerbation of EAE symptoms after the discontinuation of fingolimod administration. The administration of CKD-506 and fingolimod was discontinued on day 15 post-EAE induction. The discontinuation of CKD-506 did not affect EAE symptoms, whereas that of fingolimod exacerbated EAE symptoms (Fig. 6c). The final clinical score in the CKD-506-treated group was significantly lower than that in the vehicle group [Fig. 6d; F(4, 47) = 21.00; *p* < 0.001; one-way ANOVA]. Additionally, the symptom severity in the CKD-506-treated group on day 22 post-EAE induction was significantly lower than that in the vehicle-treated group after fingolimod was replaced with CKD-506 or vehicle on day 15 post-EAE induction [Fig. 6c; F(1, 280) = 13.47; *p* = 0.0003; two-way ANOVA]. This suggests that CKD-506 delayed the exacerbation of the clinical symptoms in mice with EAE.

### CKD-506 mitigated the fingolimod discontinuation-induced increase in the number of leukocytes and maintained the therapeutic effect on the levels of TNF-α after discontinuation

Next, the number of leukocytes and the levels of cytokines in the blood of the vehicle group were examined to evaluate the effects of CKD-506 on the peripheral immune response after drug discontinuation or substitution. Pathogenic subpopulations of immune cells from the peripheral lymphoid organs enter the CNS through blood vessels and contribute to the progression of MS. Thus, an increase in the number of leukocytes in the peripheral blood may indicate an enhanced immune response associated with the pathogenesis of MS. Lymphocytes rapidly escaping from the secondary lymphoid organ may exacerbate disease symptoms immediately after fingolimod is discontinued. The leukocyte number in the vehicle group on day 18 post-EAE induction was similar to that in the non-immunized groups. Similarly, the number of leukocytes was similar between the group in which CKD-506 was discontinued and that in which CKD-506 treatment was continued (Fig. 6e). In contrast, the leukocyte number rapidly increased in the group in which fingolimod was discontinued [Fig. 6e; F(6, 41) = 5.551; *p* < 0.001; one-way ANOVA test]. However, treatment with CKD-506 significantly mitigated the fingolimod discontinuation-induced increase in leukocyte number (Fig. 6e; T(14) = 1.815, *p* = 0.0473; unpaired *t*-test). Also, the blood cytokine analysis of the EAE mice at 18 days after induction showed that the effect of CKD-506 on the levels of TNF-α persisted even after drug discontinuation [Supplementary data; F(4, 30) = 20.72; *p* < 0.0001; one-way ANOVA].

### CKD-506 did not reduce the white blood cell (WBC), red blood cell (RBC), and platelet (PLT) counts in normal mice unlike LBH-589, a pan-HDAC inhibitor

Previous studies have demonstrated that HDAC inhibition has a suppressive effect on the bone marrow, and induces severe thrombocytopenia, leukopenia, or anemia^11^. Thus, WBCs, RBCs, and PLTs were evaluated in the blood of the normal mice to confirm the effects of CKD-506 (30 mg/kg, per oral) in comparison to LBH-589 (10 mg/kg, intraperitoneal). Following a daily administration of both drugs for 3 consecutive days, LBH-589 significantly reduced WBC and PLT counts, but not that of RBCs [Supplementary data; F(2, 12) = 179.4; *p* < 0.0001 for WBC, F(2, 12) = 153.0; *p* < 0.0001 for PLT; one-way ANOVA]. However, CKD-506 (30 mg/kg) showed no effects on the levels of these cells.

### CKD-506 alleviated depression at the pre-symptomatic stage of EAE

**Figure 7:**
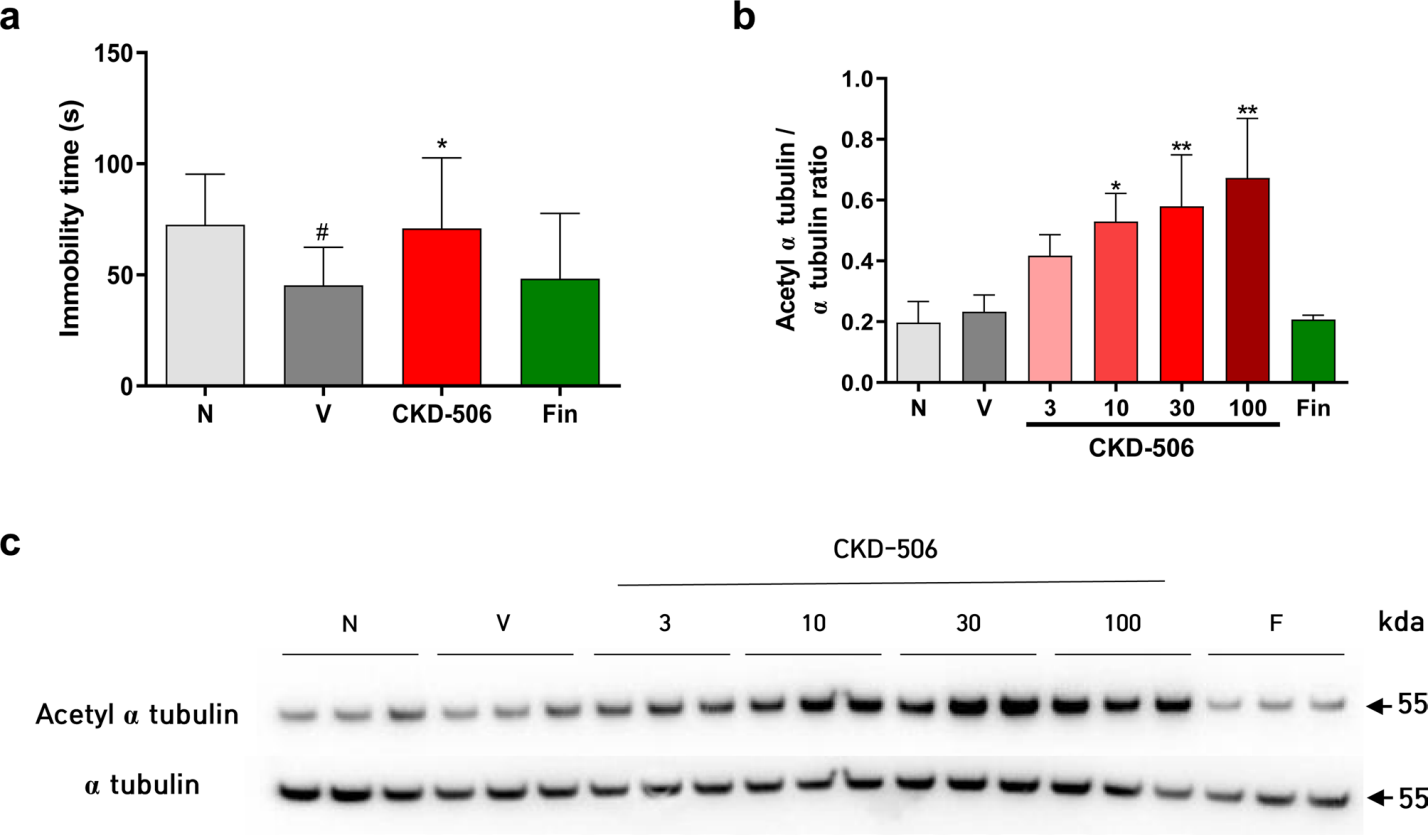
CKD-506 alleviated depression-like behavior, as evaluated using the tail suspension test. Additionally, CKD-506 upregulated the acetylated α-tubulin level in the brain. Mice were orally administered CKD-506 (30 mg/kg bodyweight) and fingolimod (0.3 mg/kg bodyweight) daily from days 6 to 8 post-myelin oligodendrocyte glycoprotein_35–55_ immunization (**a**–**c**). Tail suspension test (n = 12 per group for the non-immunized groups; n = 13 per group for the other groups) (**a**) and analysis of acetylated α-tubulin expression (n = 3 per group) (**b**) were performed during the pre-symptomatic stage on day 8 post-induction. (**c**) Protein bands of acetylated α-tubulin and α-tubulin are shown. Data are presented as mean ± SD and analyzed using one-way ANOVA, followed by Dunnett’s post-hoc test. #*p* < 0.05, non-immunized groups versus vehicle group; *p < 0.05 and **p < 0.01, drug-treated group versus vehicle group. N, non-immunized groups; V, vehicle group; Fin, fingolimod (0.3 mg/kg bodyweight)-treated group.

Clinical studies have reported that the administration of new drugs to patients with MS can induce depression. More than 50% of the patients with MS exhibit depression during their lifetime^24^. Thus, a tail suspension test (TST) was performed and the expression levels of acetylated α-tubulin and α-tubulin in the brain were examined on day 8 post-EAE induction to evaluate the CNS-penetrating ability of CKD-506 to alleviate depression-like behavior in mice with EAE during the pre-symptomatic stage. In TST, mice with EAE exhibited a decreased period of immobility, which indicates depression-like behavior in this model^25^. Moreover, the CKD-506-treated group exhibited a higher immobility latency than the vehicle group. The immobility latency value in the CKD-506-treated group was similar to that in the non-immunized groups [Fig. 7a; F(3, 47) = 3.944; *p* = 0.0137; one-way ANOVA]. The level of acetylated α-tubulin in the CKD-506 (10 mg/kg bodyweight)-treated group was significantly higher than that in the vehicle-treated group [Fig. 7b, C; F(6,14) = 9.071; *p* < 0.001; one-way ANOVA]. This suggests that CKD-506 (10 mg/kg bodyweight) could penetrate the brain. These findings demonstrate that CKD-506 could alleviate depression-like behavior by exerting therapeutic effects in the brain.

## Discussion

Over the last decade, the treatment outcomes of patients with MS have markedly improved. However, disease relapse and progression are observed in several patients. In some cases, there are limited therapeutic strategies available, which highlights the need for the continuous monitoring of the risk of developing side effects. Thus, there is a need to develop novel therapeutic strategies against MS. In this study, the potential therapeutic effects of CKD-506, a novel HDAC6-specific inhibitor, against MS were examined under various treatment regimens using the MOG_35–55_-induced EAE mouse model.

Previous studies have demonstrated that pan-HDAC inhibitors could alleviate the symptoms of several autoimmune diseases by enhancing the anti-inflammatory response^8,26^. However, pan-HDAC inhibitors have a narrow therapeutic spectrum, which limits their application as therapeutics for MS^11,26^. Compared with pan-HDAC inhibitors, HDAC6 inhibitors have a better safety profile since they predominantly acetylate cytoplasmic non-histone proteins^27,28^. Moreover, phase I clinical trials for CKD-506, a highly selective HDAC6 inhibitor, showed no significant safety issue (EudarCT number:2016-002816-42), and in this study as well, CKD-506, at the therapeutic dose, did not induce bone marrow suppression, unlike LBH-589.

CKD-506 also markedly alleviated the clinical symptoms and inhibited inflammation and demyelination in the spinal cord of the MOG_35–55_-induced EAE mouse model under both prophylactic and therapeutic regimens. The therapeutic effects of CKD-506 were comparable with those of fingolimod, which is a second-line therapeutic for MS. Currently, patients resistant to first-line therapeutics are administered fingolimod, which decreases the relapse rate by 60% within 1 year after treatment^5^.

CKD-506 directly suppressed the proliferation of pathogenic immune cells in the MOG_35–55_-stimulated splenocytes. It also decreased the infiltration of T cells and macrophages as well as the levels of pro-inflammatory cytokines in the blood or the spinal cord of EAE mice. These findings suggest that CKD-506 regulates the proliferation of the pathogenic subpopulations of immune cells and suppresses their activity by reducing the levels of pro-inflammatory cytokines such as IL-12, IL-17A, IFN-γ, IL-1β, and TNF-α in EAE. The activation and expansion of the pathogenic immune cells from the peripheral lymphoid organs are critical for the development of MS in the initial relapse process^29,30^. Previous studies have also shown that the number of pathogenic immune cells, such as certain subpopulations of T cells and macrophages, is elevated in the blood and CNS lesions of patients with MS during the relapse phase^31,32^.

The levels of IL-12, IFN-γ, and IL-17A, mainly expressed in the Th1/17 cells, and of IL-1β and TNF-α, mainly expressed in the microglia/macrophages are usually higher in the blood, CSF, and lesion sites of the patients with MS, compared with healthy individuals and those cytokines have close relations with the progression of MS^33–36^. IL-12 is an enhancer of the polarization of T cells to Th1 cells, a pathogenic Th subset that contributes to the progression of MS directly by damaging the myelin sheath of the oligodendrocytes and indirectly by enhancing the activation of potentially pathogenic macrophages through the secretion of TNF-α or IFN-γ^37^. MOG-induced symptoms and CNS lesions are alleviated in *Il12* knockout mice^38^. IFN-γ is a hallmark of Th1 cells inducing inflammation and autoimmune responses, such as those seen in MS. The effects of IFN-γ on MS pathology are controversial and disease stage dependent. However, the expression levels of IFN-γ in patients with MS are usually higher than those in healthy individuals^33^, and blocking IFN-γ significantly improved the symptoms in secondary progressive MS^39^. Thus, Lowering IFN-γ could be one treatment strategy for MS. IL17A, a key cytokine of Th17, has also demonstrated efficacy against MS in a pilot study with its antibody, sekukinumab^34^, while the blockade of IL-17A ameliorated the disease symptoms in various models of EAE mice^40,41^. Although the clinical effects of IL-1β and TNF-α are not clear since their respective antibodies show no positive response in clinical studies, various studies have suggested their important roles in the pathogenic responses of EAE mice and those seen in MS. Clinical symptoms of EAE were significantly attenuated in IL-1 receptor-deficient and IL-1β-deficient mice^35^. Decreased levels of TNF-α improved EAE symptoms by protecting the BBB permeability and inhibiting the immune cell migration in various conditions of TNF-α deficit^36^.

BBB dysfunction also contributes to the progression of EAE and MS by enabling the migration of activated pathogenic cells and toxic molecules into the brain, which induces neuronal inflammation, demyelination, and neural cell death^23^. In this study, CKD-506 protected the BBB by upregulating the expression of occludin, a major component of BBB. Occludin, a transmembrane protein, enhances the tight junction integrity by forming a scaffold with ZO-1. Previous studies have also demonstrated that the downregulation of occludin enhances BBB permeability^42^. CKD-506 downregulates the levels of ICAM-1, VCAM-1, and IP-10 in LPS-induced human peripheral blood mononuclear cells and an RA rat model^20^. Additionally, CKD-506 regulates the morphology of immune cells by rearranging tubulin and actin and consequently, inhibits their migration^20,43^. In this study, a decrease in the number of macrophages in the spinal cord also demonstrated the inhibitory effects of CKD-506 on immune cell migration. Clinical studies on natalizumab have demonstrated that the inhibition of pathogenic immune cell chemotaxis and migration into the brain decreases the risk of MS relapse^44^. These findings indicate that CKD-506 could alleviate MS by enhancing the BBB integrity and regulating the chemo-attraction and migration of immune cells.

The US Food and Drug Administration has warned that fingolimod discontinuation may exacerbate disease symptoms, which was also reported in a clinical study^45^. We found that fingolimod discontinuation rapidly exacerbated EAE symptoms and increased the number of leukocytes and levels of Th1-related cytokines such as IFN-γ in the blood after 3 days. This suggests that the rapid deterioration of EAE symptoms may be attributed to the increased escape of pathogenic T cells into the blood from the lymphoid organ immediately after fingolimod discontinuation. However, the therapeutic effects of CKD-506 were observed even after the drug was discontinued. TNF-α and IFN-γ were maintained at lower levels 3 days after drug discontinuation. However, it remains unclear as to why the efficacy of CKD-506 was maintained.

Additionally, CKD-506 delayed the exacerbation of symptoms and prevented the rapid escape of leukocytes after the discontinuation of fingolimod. The mechanisms underlying the CKD-506-mediated delay in symptom exacerbation and inhibition of rapid leukocyte escape have not been elucidated. However, the findings suggest that CKD-506 could delay the progression of EAE even when the number of active pathogenic cells markedly increased in the systemic circulation, which is a characteristic of the relapse phase of MS.

CKD-506 increased the levels of acetylated α-tubulin in the brain and alleviated the depression-like behavior at the pre-symptomatic stage of the EAE model. Currently, 50% of the patients with MS suffer from depression, which has no effective treatment. Thus, the efforts to treat MS-associated depression are on-going. Furthermore, previous studies on *Tnfar* and *Il-1β* knockout mice demonstrated that the downregulation of these cytokines alleviates depression-like behaviors^46,47^. The upregulation of these cytokines in the hypothalamus has been shown to affect emotional behavior through the upregulation of corticosterone, which is secreted from the hypothalamic–pituitary–adrenal/thyroid axis, in mice with EAE^25^. Various neurological diseases are associated with defects in axonal transport, which induce neurological defects and lead to emotional and cognitive dysfunction^17^. Moreover, studies on mice with ALS or CMT have reported that HDAC6 inhibitors reverse axonal transport defects by upregulating the acetylation of α-tubulin^48,49^. These results suggest that CKD-506 could alleviate depression by downregulating TNF-α and IL-β expression and enhancing axonal transport through the upregulation of acetylated α-tubulin in the CNS.

This study has some limitations. We did not to confirm the subpopulations of the T cells and macrophages in the blood or spinal cord as the cytokine analysis carried out was not sufficient to confirm the subpopulation of these cells; different subpopulations of T cells and macrophages have vastly different effects on the disease. Furthermore, although the efficacy of CKD-506 was maintained after its discontinuation, with the observation of a delay in symptom exacerbation, the related mechanisms have not been elucidated. Lastly, only one behavioral test was performed for the evaluation of the depression-like features in EAE mice. Animal behavioral assessment is heavily influenced by various factors. Thus, more than two types of assessments would ensure the accuracy of the results.

Summarily, this study demonstrated that CKD-506 markedly alleviates EAE in the rodent model through the regulation of the activation and proliferation of pathogenic subpopulations of T cells and M1 macrophages in the peripheral lymphoid tissue and the maintenance of BBB integrity. Additionally, the therapeutic efficacy of CKD-506 was maintained even after its discontinuation. Furthermore, CKD-506 could delay disease progression and decrease the number of activated pathogenic cells in systemic circulation. Finally, CKD-506 alleviated depression-like behavior in mice with EAE. These findings suggest that CKD-506 is a potential therapeutic strategy against MS.

## Methods

### Chemicals

CKD-506 was developed by Chong Kun Dang Pharmaceuticals (Seoul, Korea). Fingolimod was purchased from Combi block (#ST-8895). For in vivo studies, CKD-506, fingolimod, and negative control were diluted using an ethanol:Kolliphor®:saline (1:1:8) solution. The three chemicals used for the preparation of the vehicle were obtained from Sigma-Aldrich (St. Louis, MO, USA).

### Experimental animals

Female C57BL/6J mice (aged 8–9 weeks with a bodyweight of 20–24 g) were purchased from Central Lab. Animal, Inc. (SLP, Japan). The animals were maintained under a 12-h light/dark cycle and provided with food and water ad libitum. All in vivo experiments were performed in a specific pathogen-free facility in CKD Research Institute labs (Yongin, Korea). All methods with animals were performed in accordance with the relevant guidelines and regulations of the Institutional Animal Care and Use Committee of the Laboratory Animal Center at Chong-gun dang, Korea (approval number: S-20-009) and the study was carried out in compliance with the ARRIVE guidelines.

### EAE induction and symptom evaluation

EAE was induced as described previously^50^. Briefly, mice were immunized with MOG_35–55_ (1 mg/mL) in complete Freund’s adjuvant containing *Mycobacterium tuberculosis* (3 mg/mL; Hooke laboratories, Lawrence, MA, USA). The mice were subcutaneously administered the emulsion (0.1 mL) at two sites on the lower and upper back and were intraperitoneally administered pertussis toxin (130 ng; Hooke laboratories) 2 and 48 h post-immunization. The clinical symptoms in mice were scored thrice in a week on a scale of 0–5 (0 = no clinical deficit; 1 = partial tail paralysis; 2 = partial hindlimb paralysis with tail paralysis; 3 = full hindlimb paralysis; 4 = full hindlimb and partial forelimb paralysis; and 5 = dead).

### Treatment regimens

C57BL/6 J mice were randomly divided into several groups (6–24 mice/group). The non-immunized groups were used in the experiments for each treatment regimen. The treatment regimens were as follows:

A. Prophylactic regimen: Mice were orally administered CKD-506 (3, 10, 30, and 100 mg/kg bodyweight), fingolimod (0.1, 0.3, and 1 mg/kg bodyweight), or vehicle daily from day 6 to day 29 post-EAE induction (n = 6–24).
B. Therapeutic regimen: Mice were orally administered CKD-506 (30 mg/kg bodyweight), fingolimod (0.3 mg/kg bodyweight), or vehicle daily from day 15 to day 29 post-EAE induction (n = 8–9). Prior to treatment, mice were divided into each group based on their body weight and clinical score.
C. Drug discontinuation regimen: Mice were orally administered CKD-506 (30 mg/kg bodyweight), fingolimod (0.3 mg/kg bodyweight), or vehicle daily from day 6 to day 14 post-EAE induction. The clinical score was evaluated until day 29 post-EAE induction (n = 10–13).
D. Drug change regimen: Mice of one group were orally administered fingolimod (0.3 mg/kg bodyweight) from day 6 post-EAE induction. Next, fingolimod was replaced with CKD-506 (30 mg/kg bodyweight) from day 15 to day 29 post-EAE induction (n = 10).
E. Treatment regimen to evaluate the mechanism of action: Mice were orally administered CKD-506 and fingolimod following the same schedule as A, B, C, and D mentioned above until day 18 or 21 for examination of the leukocyte number, cytokine levels, BBB permeability, and protein expression (acetyl-α tubulin and occludin) or until day 29 for histological, IHC, and cytokine analyses.

### Histological analysis

Mice were anesthetized with isoflurane (Hana Pharm Inc., Korea) and transcardially perfused with saline (3 mL). The spinal cord was excised and fixed with 10% neutral-buffered formalin at room temperature. The samples were cut into pieces, processed, embedded in paraffin, and sectioned to 4-μm thick sections using a sliding microtome. The sections were stained with H&E (BBC Biochemical, Mount Vernon, WA, USA) or LFB (VitroVivo Biotech, # VB-3006) and observed under a light microscope. The severity of immune cell infiltration with the H&E-stained sections was assessed and scored in the meninges, parenchyma, and vessels of the spinal cord by two histologists who were blind to the treatments and grouping. Then, the scores in the three regions were added. The scoring to determine the histological score was performed as follows: for meninges and parenchyma, no infiltrating cells = 0; few infiltrating cells = 1, numerous infiltrating cells = 2, and widespread infiltration = 3; for vessels, no cuffed vessel = 0; one or two cuffed vessels per section = 1, three to five cuffed vessels per section = 2, and more than five cuffed vessels per section = 3. Additionally, the demyelinated and total areas of white matter were measured using ImageJ software. The percentage of demyelination was calculated as follows: demyelinated area (%) = [(demyelinated area in white matter) / (total white matter area) × 100].

### IHC analysis

The paraffin-embedded sections (4 μm) of the spinal cords were mounted on glass slides. IHC analysis of the sections was performed using an automated slide preparation system (Benchmark XT; Ventana Medical Systems Inc., Tucson, AZ, USA). Deparaffinization, epitope retrieval, and immunostaining were performed using cell conditioning solutions and the BMK ultraVIEW diaminobenzidine detection system (Ventana Medical Systems Inc., Tucson, AZ), following the manufacturer’s instructions. The tissue sections were stained with anti-CD3 (#ab16669, Abcam), anti-CD68 (#ab125212, Abcam), and anti-PAX5 (#ab109443, Abcam) primary antibodies. The positive signals were amplified using ultraVIEW copper. The sections were counterstained with hematoxylin and bluing reagent. The number of CD3-, CD68-, and PAX5-immunoreactive cells in the white matter and pia mater of the spinal cord was examined using a light microscope and normalized to the total area of white matter and pia mater.

### Cytokine analysis

Mice were anesthetized with isoflurane and the blood sample was collected from the inferior vena cava on day 29 post-EAE induction. Systemic perfusion was performed by infusing saline (3 mL) into the left ventricle of the heart through the opened vena cava. Lumbar spinal cord (L3–L5) was excised and homogenized in ice-cold homogenizing buffer (PBS containing 0.1% Triton X-100 and 1% protease inhibitor mixture). The blood and homogenized spinal cord samples were then centrifuged at 12,000 rpm and 4 °C for 15 min. The levels of cytokines in the supernatant were measured using the multiplex cytokine kits (Millipore, #MHSTCMAG-70 K), following the manufacturer’s instructions. The protein concentration was measured using bicinchoninic acid protein assay reagents (Thermo Scientific Inc., # VC297454). The cytokine levels in the spinal cord homogenate were normalized with the protein concentration.

### Protein extraction and immunoblotting

Proteins extracted from the spinal cord (L3–L5) and the brain of mice with EAE were resolved using pre-made SDS-PAGE gels (NuPAGE® Bis–Tris Precast Gels, Invitrogen). The resolved proteins were transferred onto a PVDF membrane and probed with the following primary antibodies: anti-acetyl α-tubulin (1:5,000, #5335, Cell Signaling Technology), anti-α-tubulin (1:2,000, #2144, Cell Signaling Technology), anti-occludin (1:1,000, #40-4700, Invitrogen), and anti-β-actin (1:5,000, #A5441, Cell Signaling Technology). Next, the membrane was incubated with HRP-conjugated anti-rabbit IgG (1:5,000, #7074S, Cell Signaling Technology) and anti-mouse IgG (1:5,000, #7076 S, Cell Signaling Technology) secondary antibodies. Immunoreactivity was visualized using chemiluminescence (RPN2235, GE healthcare) with the gel documentation system.

### Proliferation assays and cytokine analysis

Single-cell suspensions were prepared from the spleen of EAE mice collected on day 12 post-EAE induction. The cells (2 × 10^5^ cells/well) were cultured in 96-well plates in the presence of the MOG peptide (10, 20, and 40 μg/mL; Hooke laboratories). The samples were processed in duplicate plates and the experiments were repeated four times. After 48 h, the cells in one plate were incubated with CCK8 solution and the absorbance at 500 nm was measured after 3 h. The culture in the other plate was centrifuged at 12,000 rpm and 4 °C to obtain the supernatant for cytokine analysis. The cytokine level in the supernatant was evaluated as mentioned above.

### Analysis of BBB permeability

BBB permeability was evaluated using EB dye as previously described^51^. On day 21 post-EAE induction, the mice were intravenously injected with EB dye (2% in PBS; 4 mL/kg bodyweight; Sigma), which was allowed to circulate for 1 h. The mice were then perfused with saline (25 mL) and the spinal cord was excised and weighed. The tissues were homogenized in formamide (0.3 mL; Sigma Aldrich) and incubated overnight at 55 °C for EB extraction. The absorbance value of the EB in the extracts was measured at 500 nm. Additionally, the absorbance value was adjusted to the volume of EB using the standard curve prepared using different concentrations of EB. The final value was represented by dividing the volume of EB in the tissue by the tissue weight.

### Complete blood count (CBC)

CBC was measured using a CBC analyzer (Sysmex, #XN-1000 V) to determine the changes in the number of leukocytes in the vehicle group subjected to the drug discontinuation regimen and the levels of CKD-506 and fingolimod in the blood of the vehicle groups subjected to the drug discontinuation and drug change regimens.

### TST

Depression behavior in mice was evaluated using TST. The mice were suspended by attaching the tip of the tail with adhesive tape (approximately 1 cm) 10-cm above the floor. To assess the depression-like behavior, immobility time (in seconds) of mice was recorded and evaluated for 5 min. To reduce the experimental bias, two analysts performed the test blindly.

### Statistical analysis

The details of the statistical analysis carried out, including statistical tests, exact p-values, and sample size are provided in the Results section and figure legends. Briefly, the means between multiple experimental groups were analyzed using Kruskal–Wallis statistic, one-way or two-way ANOVA, followed by post-hoc Dunnett’s, Tukey’s, or Bonferroni’s test. Student’s *t*-test was performed to compare the means of two groups. The differences were considered significant at *p* < 0.05. Data are expressed as mean ± SD. All statistical analyses were performed using GraphPad Prism (ver 9.0).

## Supplementary Information

Supplementary Information 1.

Supplementary Information 2.

## Data Availability

The datasets used and/or analyzed during the current study are available from the corresponding author upon reasonable request.
