# Supplementary material for: CKD-506: A novel HDAC6-selective inhibitor that exerts therapeutic effects in a rodent model of multiple sclerosis
Source: Sci Rep. 2021 Jul 14;11:14466. doi: 10.1038/s41598-021-93232-6 (PMC8280216; doi:10.1038/s41598-021-93232-6)
Supplement: Supplementary file 1 — Supplementary Information 1. [file 41598_2021_93232_MOESM1_ESM.docx]

**CKD-506: A novel HDAC6-selective inhibitor that exerts therapeutic effects in a rodent model of multiple sclerosis**

**Daekwon Bae^1, 3#^, Ji-Young Lee^1^, Nina Ha^3^, Jinsol Park^3^, Jiyeon Baek^3^, Donghyeon Suh^3^, Hee seon Lim^1^, Soo Min Ko^1^, Taehee Kim^1^, Da Som Jeong^1^ and Woo-chan Son^2*^**

^1^ Department of Medical Science, Asan Medical Institute of Convergence Science and Technology, Asan Medical Center, University of Ulsan College of Medicine, Seoul 05505, Republic of Korea

^2^ Department of Pathology, University of Ulsan College of Medicine, Asan Medical Center, Seoul 05505, Republic of Korea

^3^ Department of Pharmacology, CKD Research Institute, CKD Pharmaceutical Co, Yongin, 16995, Republic of Korea

List of Supplementary Materials

Supplemental methods and results

Supplementary data 1. Flow cytometric analysis of the spinal cord of EAE mice

Supplementary data 2. Flow cytometric analysis of the blood of EAE mice

Supplementary data 3. 52 kda- and 70 kda-sized occludin/β-actin ratio in the mouse spinal cord

Supplementary data 4. Pro-inflammatory cytokine levels of the EAE mice under the therapeutic regimen.

Supplementary data 5. Pro-inflammatory cytokine levels of the EAE mice in the drug discontinuation and drug change experiments.

Supplementary data 6. Blood cell count in non-immunized normal mice following CKD-506 or LBH-589 administration.

**Supplemental Methods and results:**

**Flow cytometric analysis of the blood and spinal cord of EAE mice**

C57BL/6 (n = 3 or 4 per group) mice were orally administered CKD-506 (30 mg/kg bodyweight) daily from day 6 post-myelin oligodendrocyte glycoprotein_35–55_ immunization; single-cell suspensions were obtained from the blood or spinal cord of experimental autoimmune encephalitis (EAE) mice upon autopsy on day 21.

The spinal cord analysis was performed by staining with FITC-conjugated anti-mouse CD4 (eBioscience, San Diego, CA, USA), PE-conjugated anti-mouse CD11b (eBioscience), and APC-conjugated anti-mouse CD45 (eBioscience) (Supplementary data 1). Meanwhile, the blood analysis was performed by staining using BV421-conjugated anti-mouse CD3 (eBioscience), and Alexafluor488-conjugated anti-mouse CD8 (eBioscience) (Supplementary data 2).

**
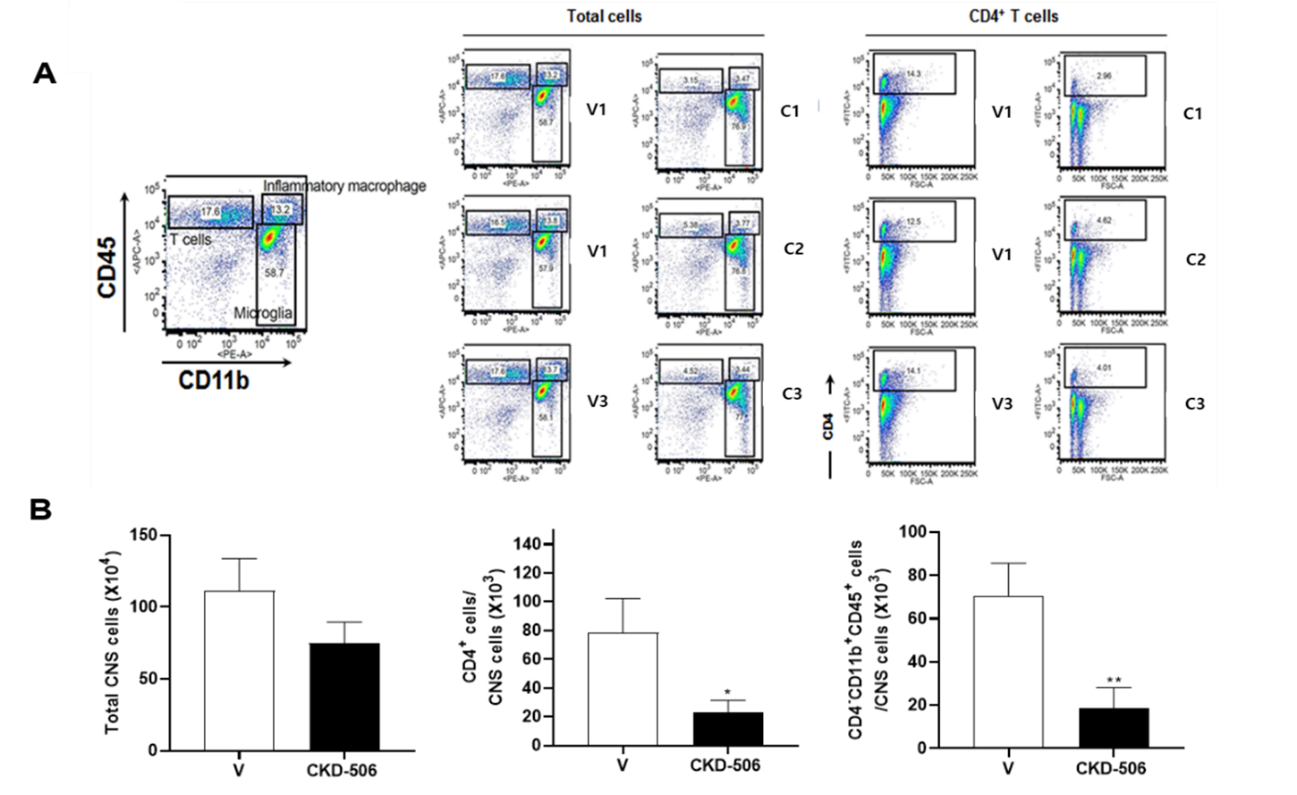
**

Supplementary data 1. Flow cytometric analyses of the spinal cord isolated from myelin oligodendrocyte glycoprotein_35–55_ (MOG_35–55_)-induced experimental autoimmune encephalitis (EAE) mice. C57BL/6 (n = 3 per group) mice were orally administered CKD-506 (30 mg/kg bodyweight) daily from day 6 post-MOG_35–55_ induction and the proportions of CD4^+^ T cells and CD4^-^CD11b^+^CD45^+^ macrophages/microglia were analyzed on day 21 post-induction (A, B). Graphs and representative multi-color flow-cytometric gating analysis images (A), proportion of CNS cells, the ratio of CD4^+^ T cells and CD4^-^CD11b^+^CD45^+^ macrophage/microglia (B). Data are presented as mean ± SD; one-way ANOVA, followed by Dunnett’s post-hoc test. *p < 0.05, **p < 0.01, drug-treated group vs. vehicle group. V, vehicle group; C or CKD, CKD-506 (30 mg/kg)-treated group.


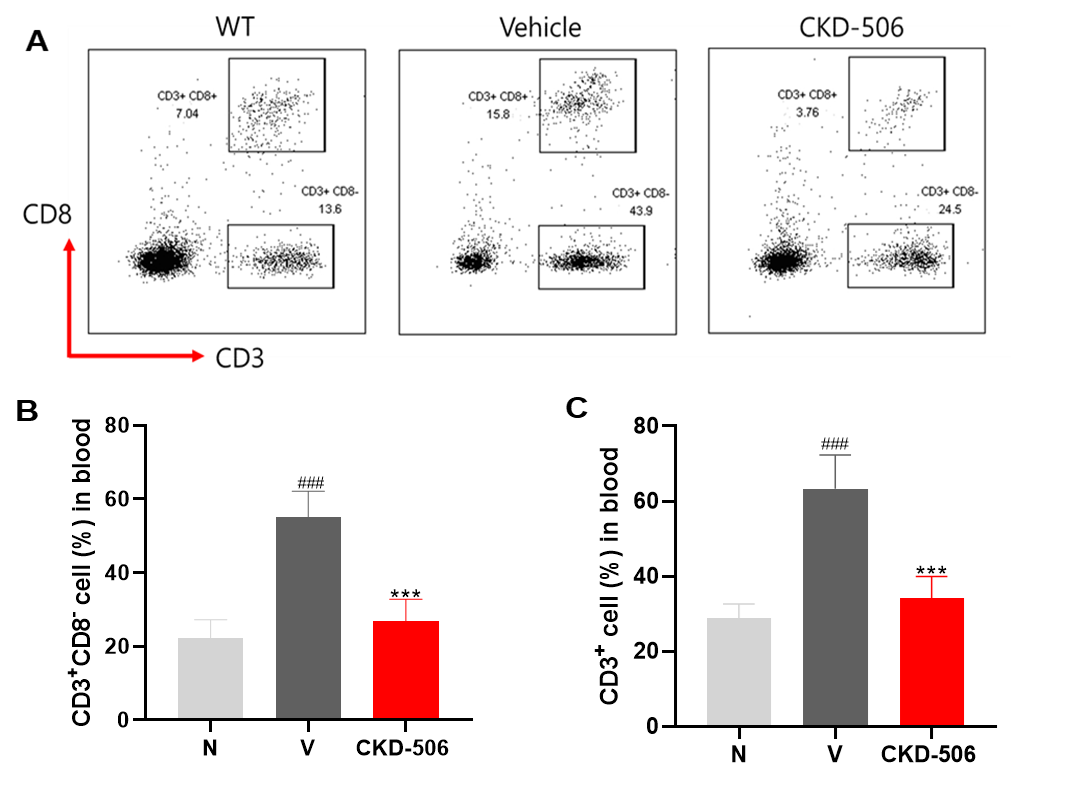


Supplementary data 2. Flow cytometric analyses of blood isolated from myelin oligodendrocyte glycoprotein_35–55_ (MOG_35–55_)-induced experimental autoimmune encephalitis (EAE) mice. C57BL/6 (n = 4 per group) mice were orally administered CKD-506 (30 mg/kg bodyweight) daily from day 6 post- MOG_35–55_ immunization and the proportions of CD3^+^ and CD3^+^CD8^-^ T cells were analyzed in their blood on day 21 post-induction (A–C). Graphs and representative multi-color flow-cytometric gating analysis images (A), proportion of CD3^+^CD8^-^ T cells (B), proportion of CD3^+^ cells (C). Data are presented as mean ± SD; one-way ANOVA, followed by Dunnett’s post-hoc test. ###p < 0.001, non-immunized groups vs. vehicle group; ***p < 0.001, drug-treated group vs. vehicle group. N, non-immunized groups; V, vehicle group.

**52 kda- and 70 kda-sized occludin/β-actin ratio in the EAE mouse spinal cord**

C57BL/6 (n = 8 per each group for the non-immunized and fingolimod-treated groups; n = 14 per each group for the other groups) mice were orally administered CKD-506 (10, 30, or 100 mg/kg bodyweight) and fingolimod (0.3 mg/kg) daily from day 6 post-myelin oligodendrocyte glycoprotein_35–55_ immunization and proteins were obtained from the spinal cord of EAE mice upon autopsy on day 21 post-induction to evaluate occludin expression. Protein extraction and immunoblotting were performed according to the same procedure presented in the Methods section of the manuscript.

**
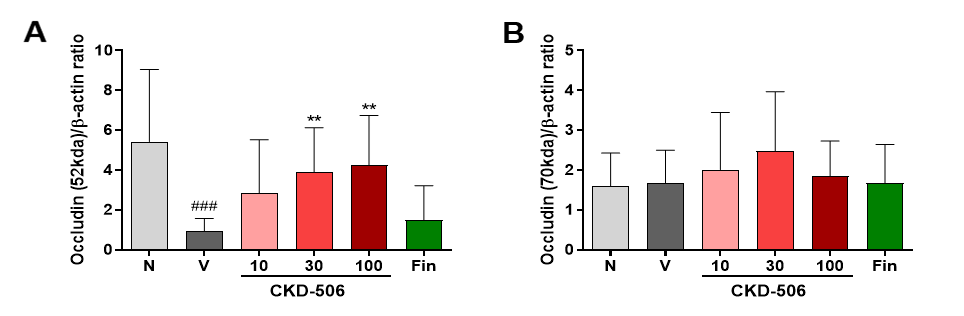
**

Supplementary data 3. The 52 kda- and 70 kda-sized occludin/β-actin ratios of the spinal cords isolated from myelin oligodendrocyte glycoprotein_35–55_ (MOG_35–55_)-induced experimental autoimmune encephalitis (EAE) mice. C57BL/6 (n = 8 per group for the N and Fin group; n = 14 per group for the other groups) mice were orally administered CKD-506 (10, 30, or 100 mg/kg bodyweight) and fingolimod (0.3 mg/kg) daily from day 6 post- MOG_35–55_ induction and the 52 kda- and 70 kda-sized occludin/β-actin ratios of their spinal cords were analyzed on day 21 post-induction (A, B). 52 kda-sized occludin/β-actin ratio (A), 70 kda-sized occludin/β-actin ratio (B). Data are presented as mean ± SD; one-way ANOVA, followed by Dunnett’s post-hoc test. ###p < 0.001, non-immunized groups vs. vehicle group; **p < 0.01, drug-treated group vs. vehicle group. N, non-immunized group; V, vehicle group; Fin, fingolimod (0.3 mg/kg)-treated group.

**Pro-inflammatory cytokine levels of EAE mice under the therapeutic regimen**

C57BL/6 (n = 7 per group) mice were orally administered CKD-506 (30 mg/kg bodyweight) and fingolimod (0.3 mg/kg) daily from day 15 post-myelin oligodendrocyte glycoprotein_35–55_ immunization. Blood was collected from the inferior vena cava on day 29 post-induction to analyze cytokine expression. Serum isolation for blood and cytokine analyses was performed according to the procedure in the Methods section of the manuscript.

**
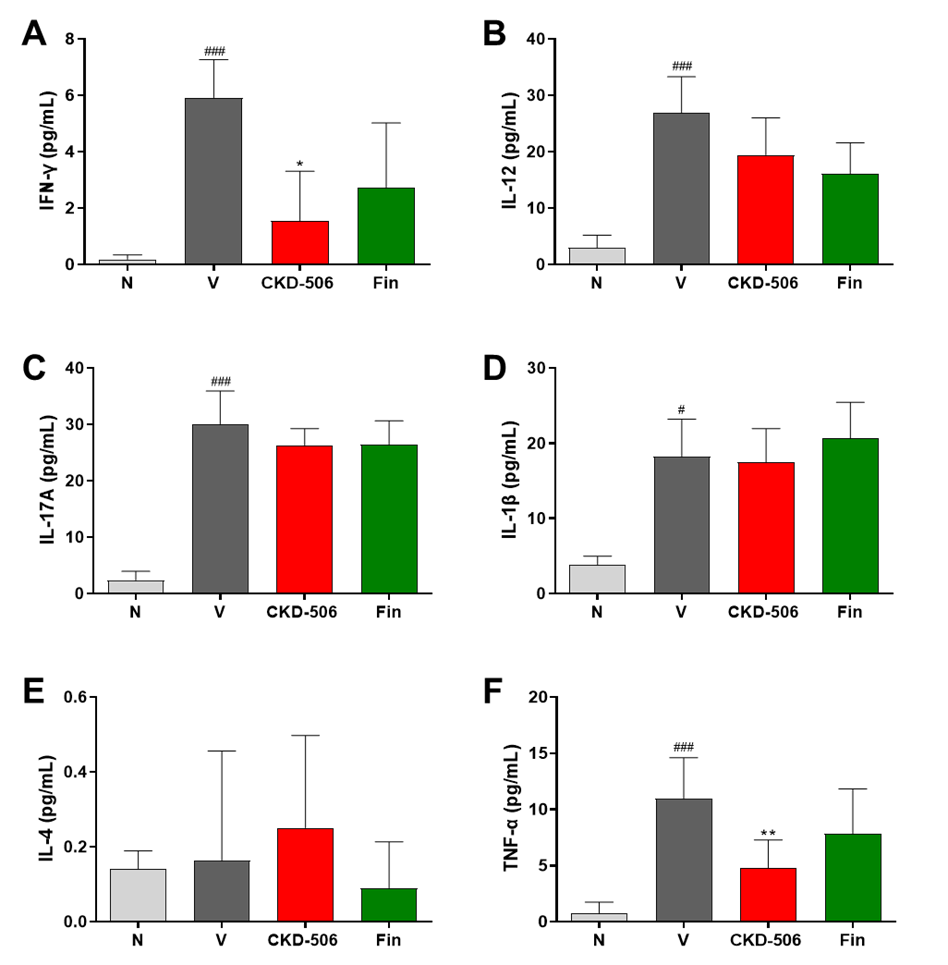
**

Supplementary data 4. Pro-inflammatory cytokine levels of the blood isolated from myelin oligodendrocyte glycoprotein_35–55_ (MOG_35–55_)-induced EAE mice treated with the therapeutic regimen. C57BL/6 (n = 7 per group) mice were orally administered CKD-506 (30 mg/kg bodyweight) and fingolimod (0.3 mg/kg) daily from day 15 post-MOG_35–55_ immunization. Pro-inflammatory cytokine levels were analyzed on day 29 post-induction (A-F). IFN-γ (A), IL-12 (B), IL-17A (C), IL-1β (D), IL-4 (E) TNF-α 70 (F). Data are presented as mean ± SD; one-way ANOVA by Dunnett’s post-hoc test for IL-12 and TNF-α and Kruskal-Wallis test by Dunnett’s post-hoc test for IFN-γ, IL-17A, IL-1β, and IL-4; #p < 0.05, ###p < 0.001, non-immunized groups vs. vehicle group; *p < 0.05, **p < 0.01, drug-treated group vs. vehicle group. N, non-immunized groups; V, vehicle-treated group; Fin, fingolimod (0.3 mg/kg)-treated group.

**Pro-inflammatory cytokine levels of the EAE mice in the drug discontinuation and drug change experiments.**

C57BL/6 (n = 7 per group) mice were treated with CKD-506 (30 mg/kg bodyweight) and fingolimod (0.3 mg/kg bodyweight) from days 6 to 14 post-induction. Treatment was discontinued or changed from fingolimod to CKD-506 on day 15 post-induction. Blood was collected from the inferior vena cava on day 18 post-induction to analyze cytokine expression. Serum isolation from blood and cytokine analysis were performed according to the procedure in the Methods section of the manuscript.

**
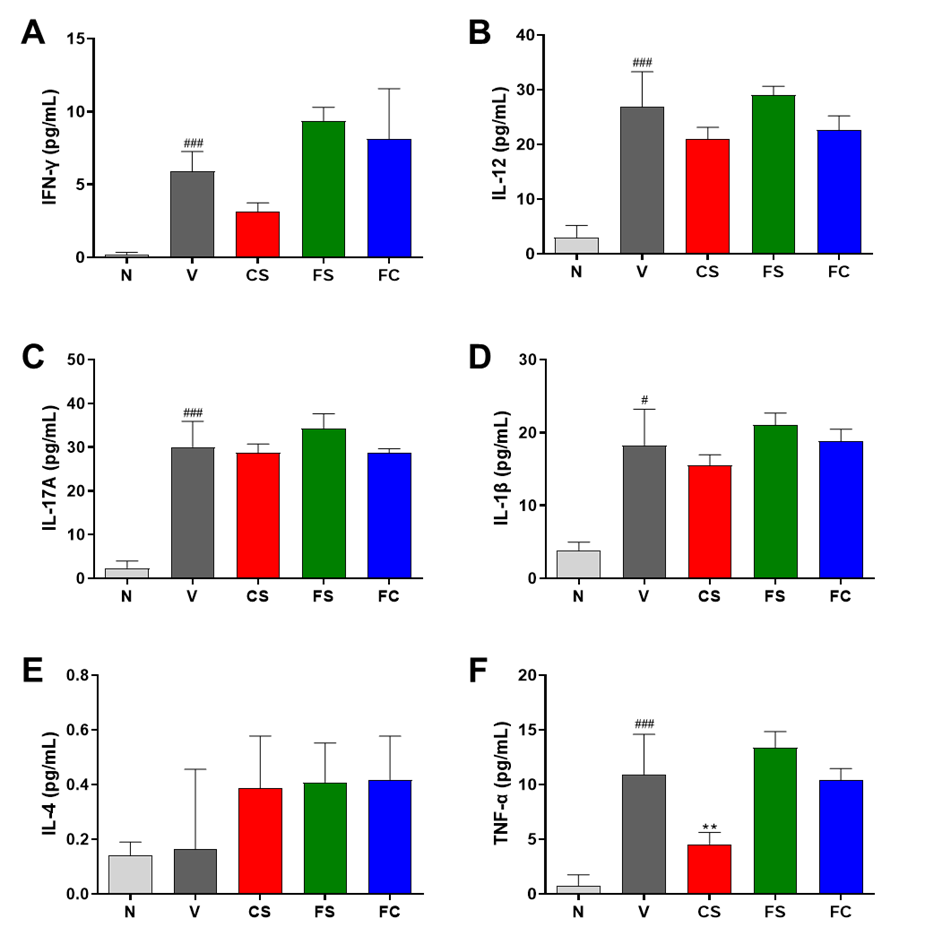
**

Supplementary data 5. Pro-inflammatory cytokine levels of the blood isolated from myelin oligodendrocyte glycoprotein_35–55_ (MOG_35–55_)-induced EAE mice in the drug discontinuation and drug change experiments. C57BL/6 (n = 7 per group) mice were treated with CKD-506 (30 mg/kg bodyweight) and fingolimod (0.3 mg/kg bodyweight) from days 6 to 14 post-induction. Treatment was discontinued or changed from fingolimod to CKD-506 on day 15 post-induction. Pro-inflammatory cytokine levels were analyzed on day 18 post-induction (A-F). IFN-γ (A), IL-12 (B), IL-17A (C), IL-1β (D), IL-4 (E) TNF-α 70 (F). Data are presented as mean ± SD; one-way ANOVA by Dunnett’s post-hoc test for IL-12 and TNF-α and Kruskal-Wallis test by Dunnett’s post-hoc test for IFN-γ, IL-17A, IL-1β, and IL-4; #p < 0.05, ###p < 0.001, non-immunized groups vs. vehicle group; **p < 0.01, drug-treated group vs. vehicle group. N, non-immunized groups; V, vehicle group; CS, CKD-506-treated group in which dosing of CKD-506 was discontinued from day 15 post-induction; FS, fingolimod-treated group in which dosing of fingolimod was discontinued from day 15 post-induction; FC, the group in which fingolimod was replaced with CKD-506 on day 15 post-induction.

**Blood cell count in non-immunized normal mice following CKD-506 or LBH-589 administration.**

C57BL/6 (n =5 per group) were daily treated with CKD-506 (30 mg/kg bodyweight, orally) and LBH-589 (10 mg/kg bodyweight, intraperitoneal) for 3 days. Then, blood was collected from the inferior vena cava at 1 h post-last administration to determine the cell count. The complete blood count was analyzed according to the procedure in the Methods section of the manuscript.

**
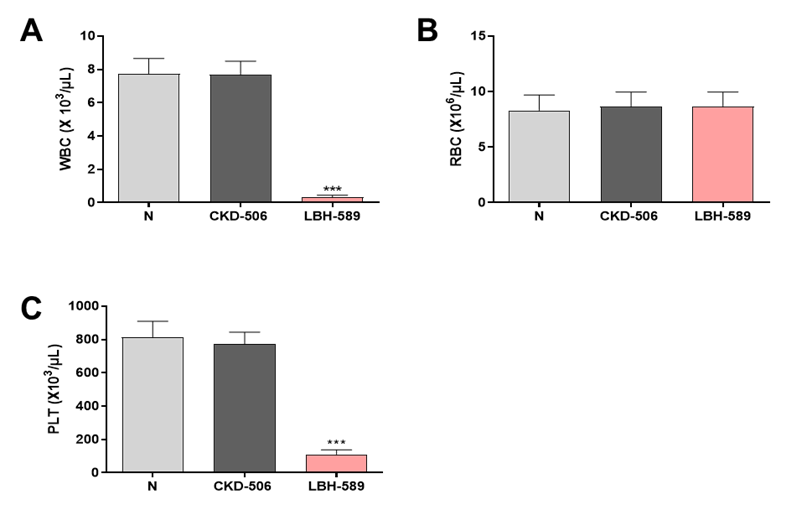
**

Supplementary data 6. White blood cell (WBC), red blood cell (RBC), and platelet (PLT) counts of non-immunized normal mice. C57BL/6 (n =5 per group) mice were daily treated with CKD-506 (30 mg/kg bodyweight, per oral) and LBH-589 (10 mg/kg bodyweight, intraperitoneal) for 3 days. Then, blood counts of WBCs, RBCs, or PLTs were analyzed 1 h post-last administration (A-C). WBC (A), RBC (B), PLT (C). Data are presented as mean ± SD; one-way ANOVA by Dunnett’s post-hoc test; ***p < 0.001, drug-treated group vs. non-immunized groups. N, non-immunized group
