# Supplementary material for: CKD-506: A novel HDAC6-selective inhibitor that exerts therapeutic effects in a rodent model of multiple sclerosis
Source: Sci Rep. 2021 Jul 14;11:14466. doi: 10.1038/s41598-021-93232-6 (PMC8280216; doi:10.1038/s41598-021-93232-6)
Supplement: Supplementary file 2 — Supplementary Information 2. [file 41598_2021_93232_MOESM2_ESM.pptx]

## Slide 1
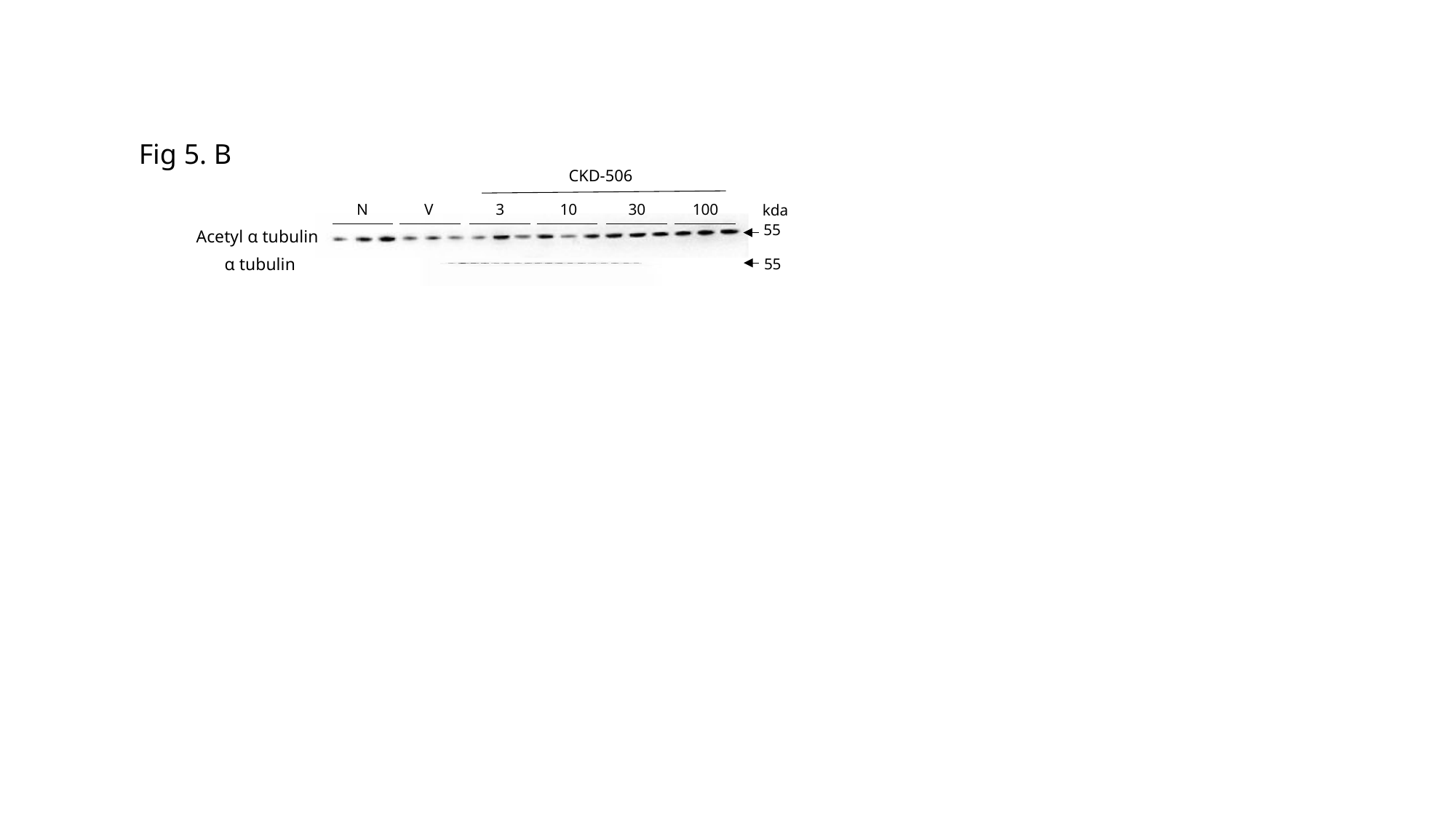

Fig 5. B
CKD-506
10
30
100
V
3
N
Acetyl α tubulin
α tubulin
kda
55
55

## Slide 2
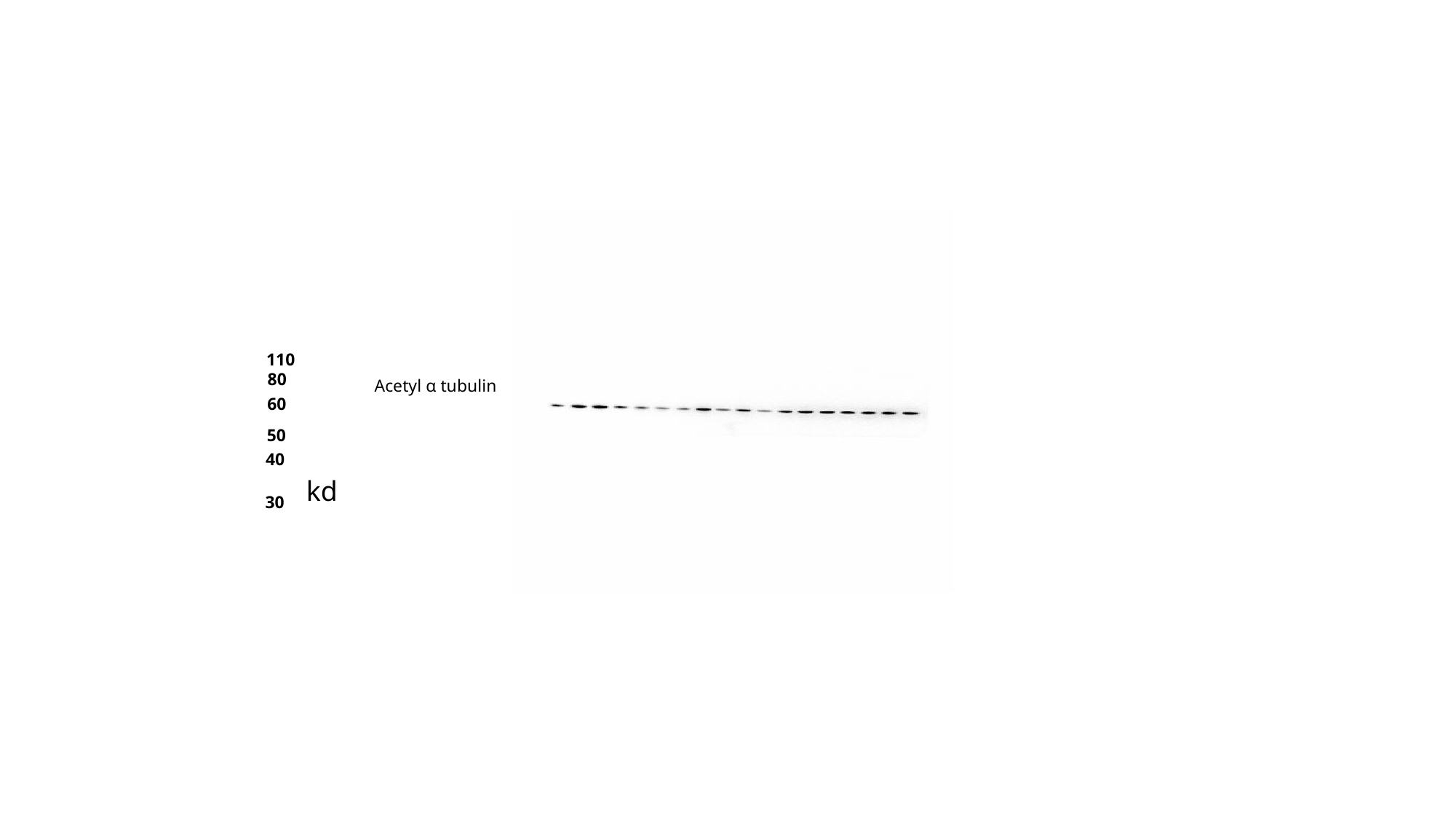

110
80
Acetyl α tubulin
60
50
40
kd
30

## Slide 3
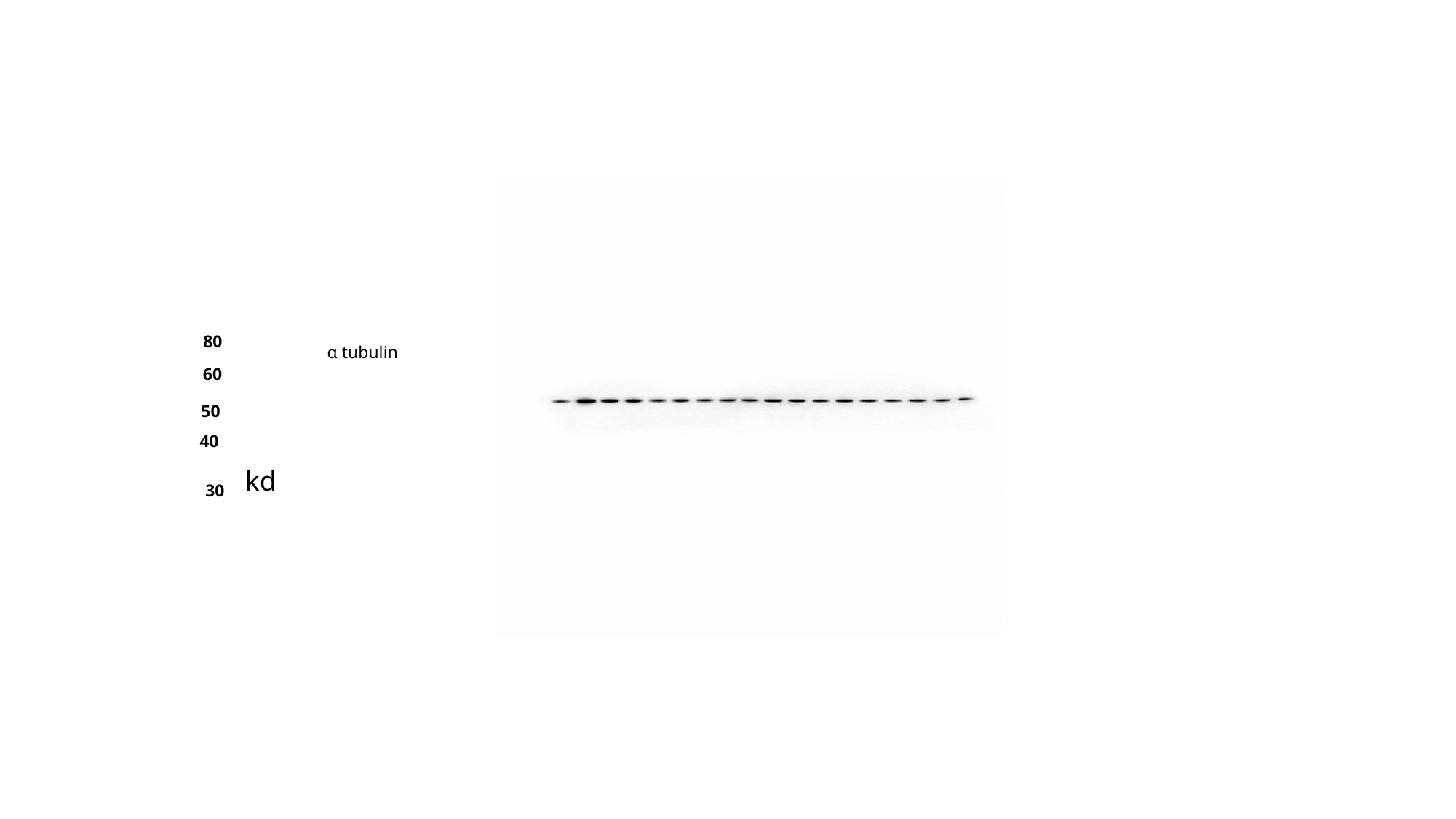

80
α tubulin
60
50
40
kd
30

## Slide 4
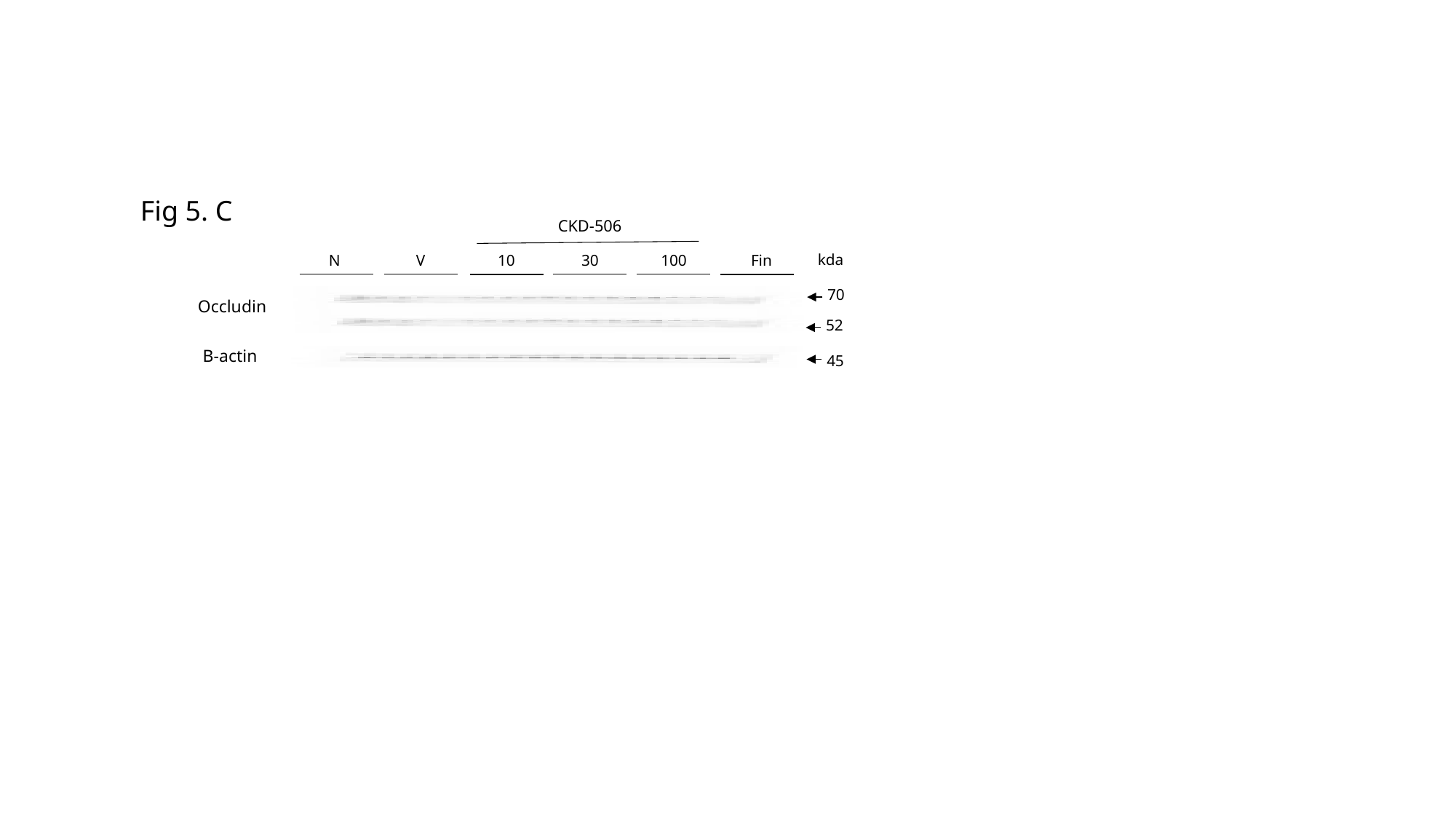

Fig 5. C
CKD-506
kda
Fin
30
100
V
10
N
70
Occludin
52
Β-actin
45

## Slide 5
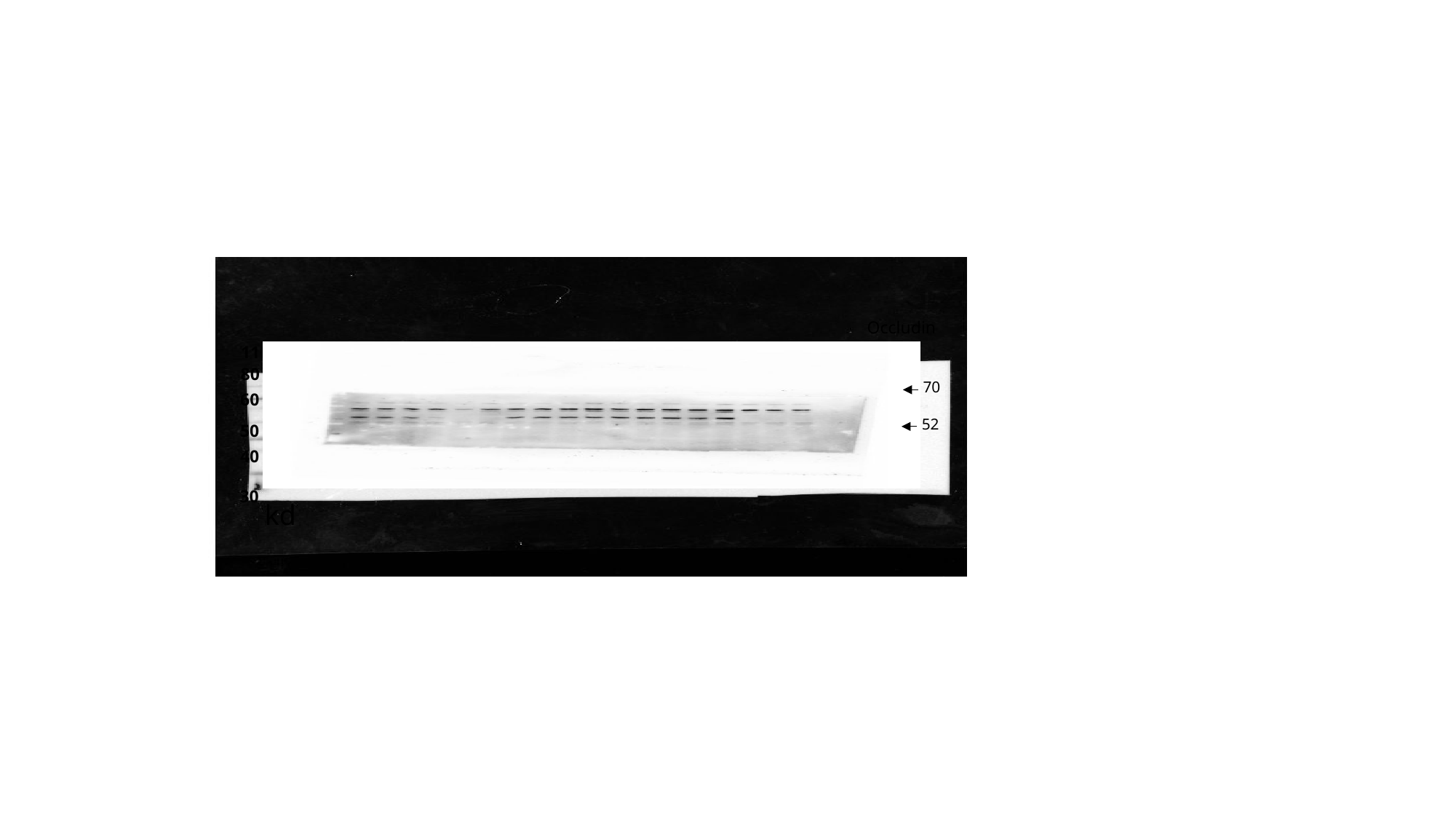

Occludin
110
80
70
60
52
50
40
30
kd

## Slide 6
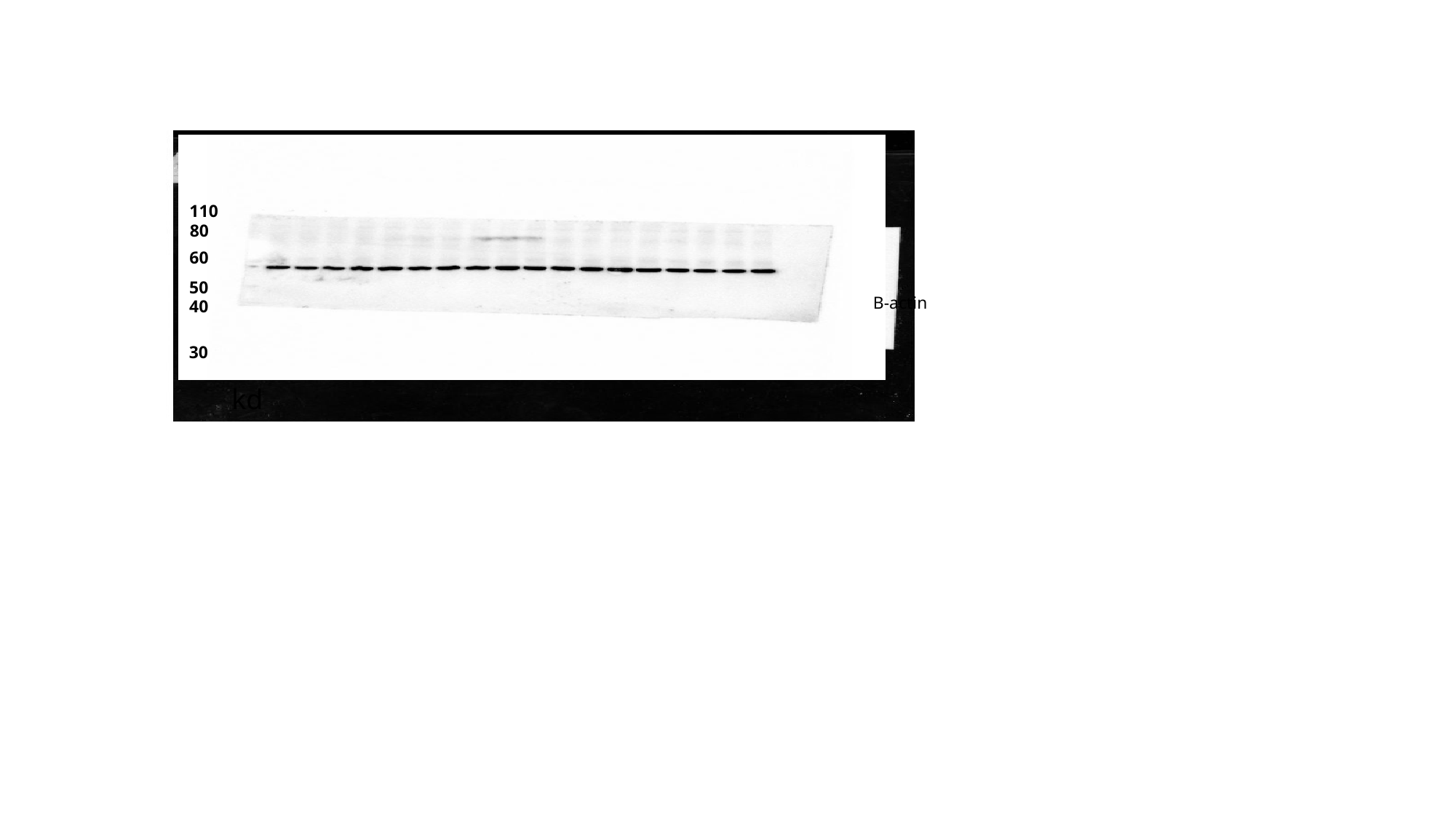

110
80
60
50
Β-actin
40
30
kd

## Slide 7
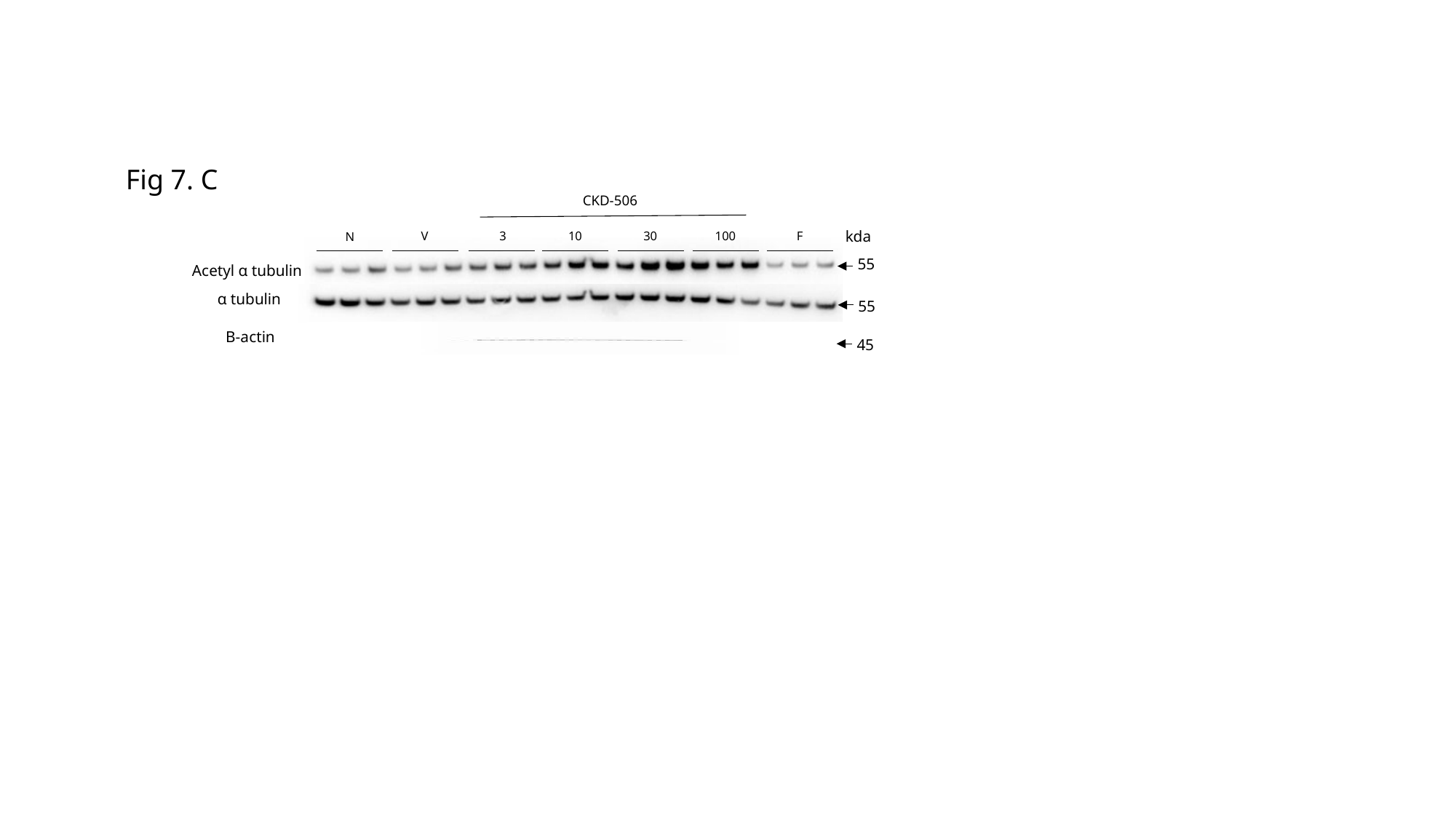

Fig 7. C
CKD-506
F
3
10
30
100
V
N
Acetyl α tubulin
α tubulin
kda
55
55
Β-actin
45

## Slide 8
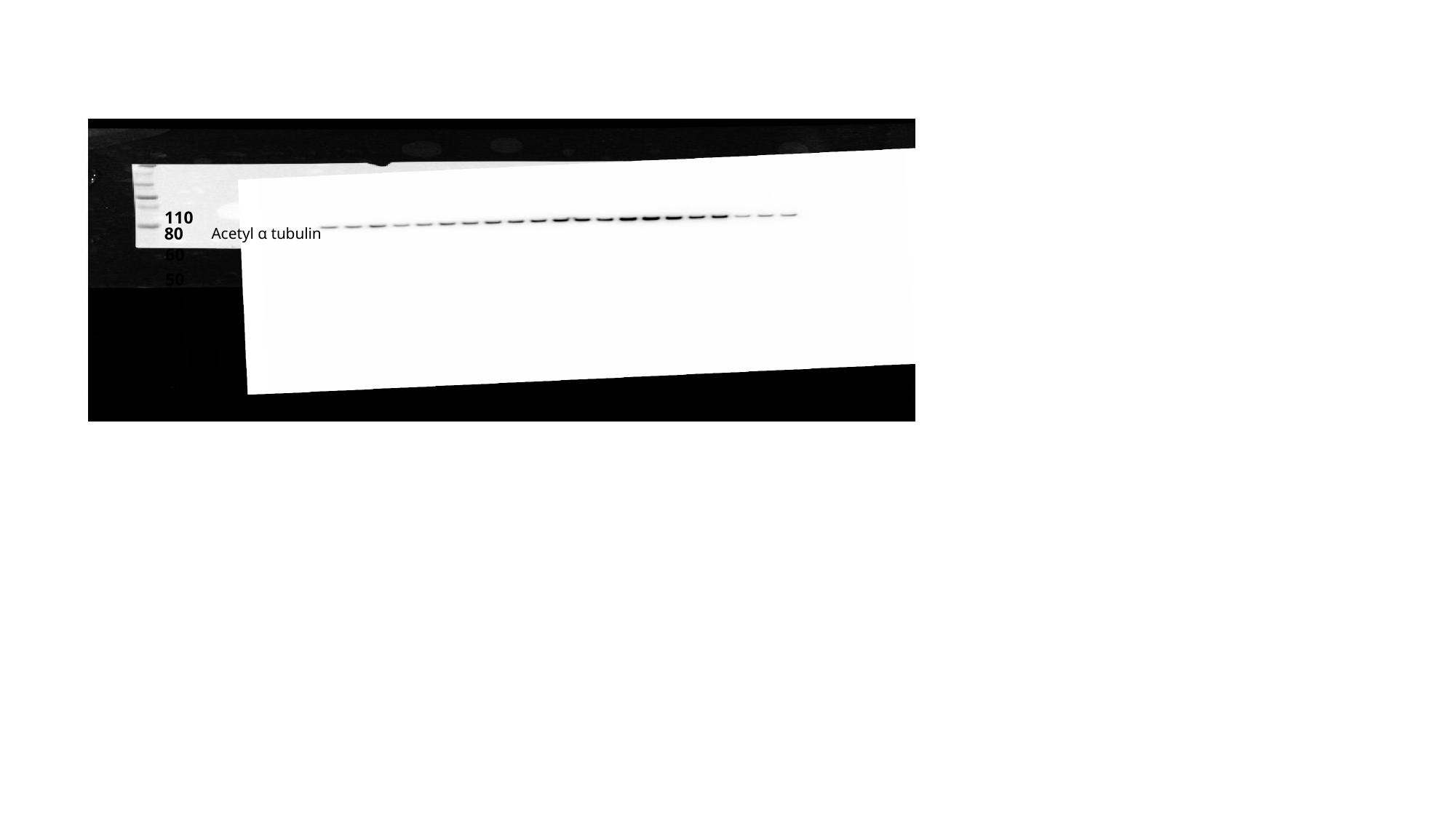

110
80
Acetyl α tubulin
60
50
40
30
kd

## Slide 9
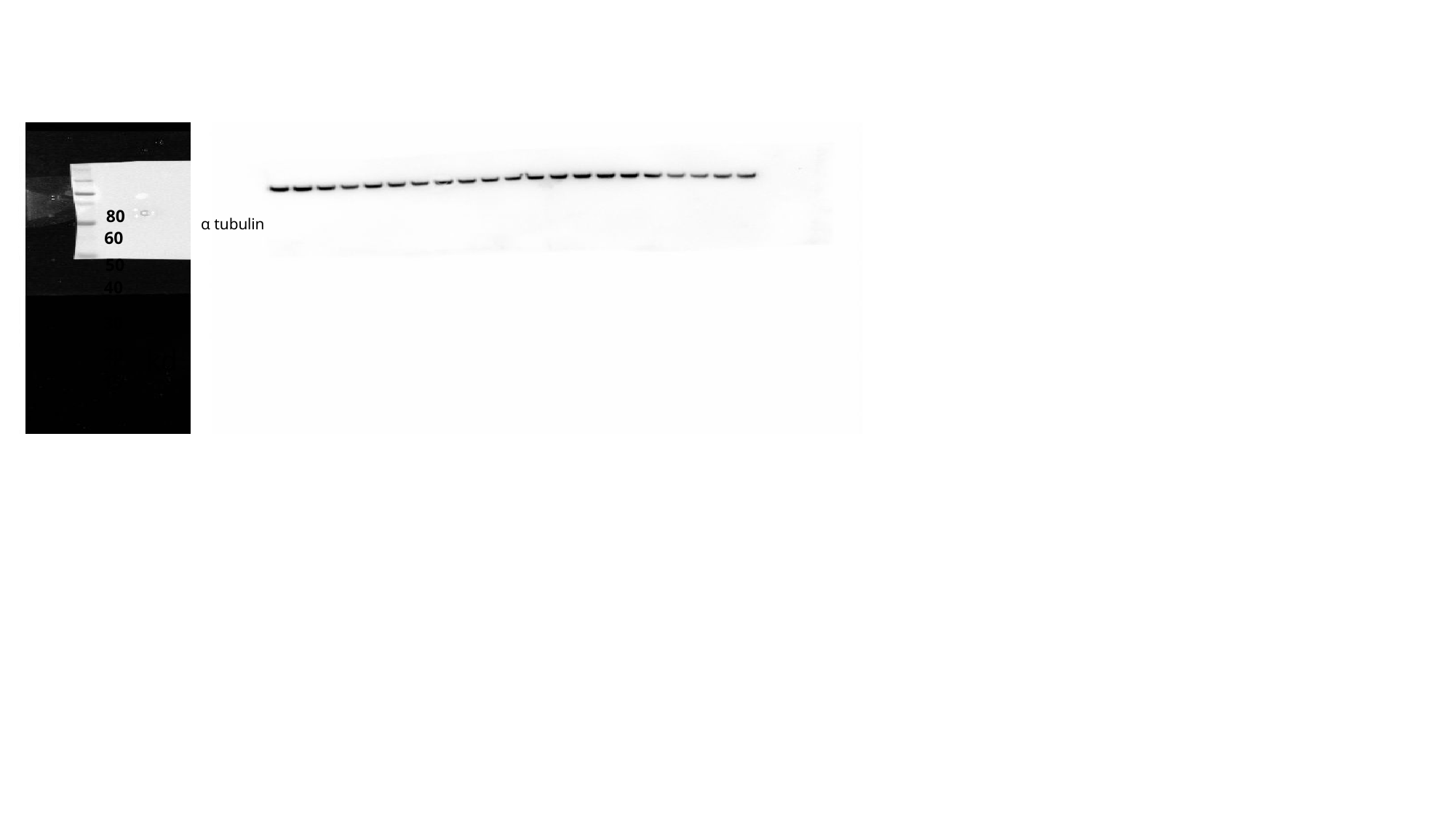

80
α tubulin
60
50
40
30
kd
20
15

## Slide 10
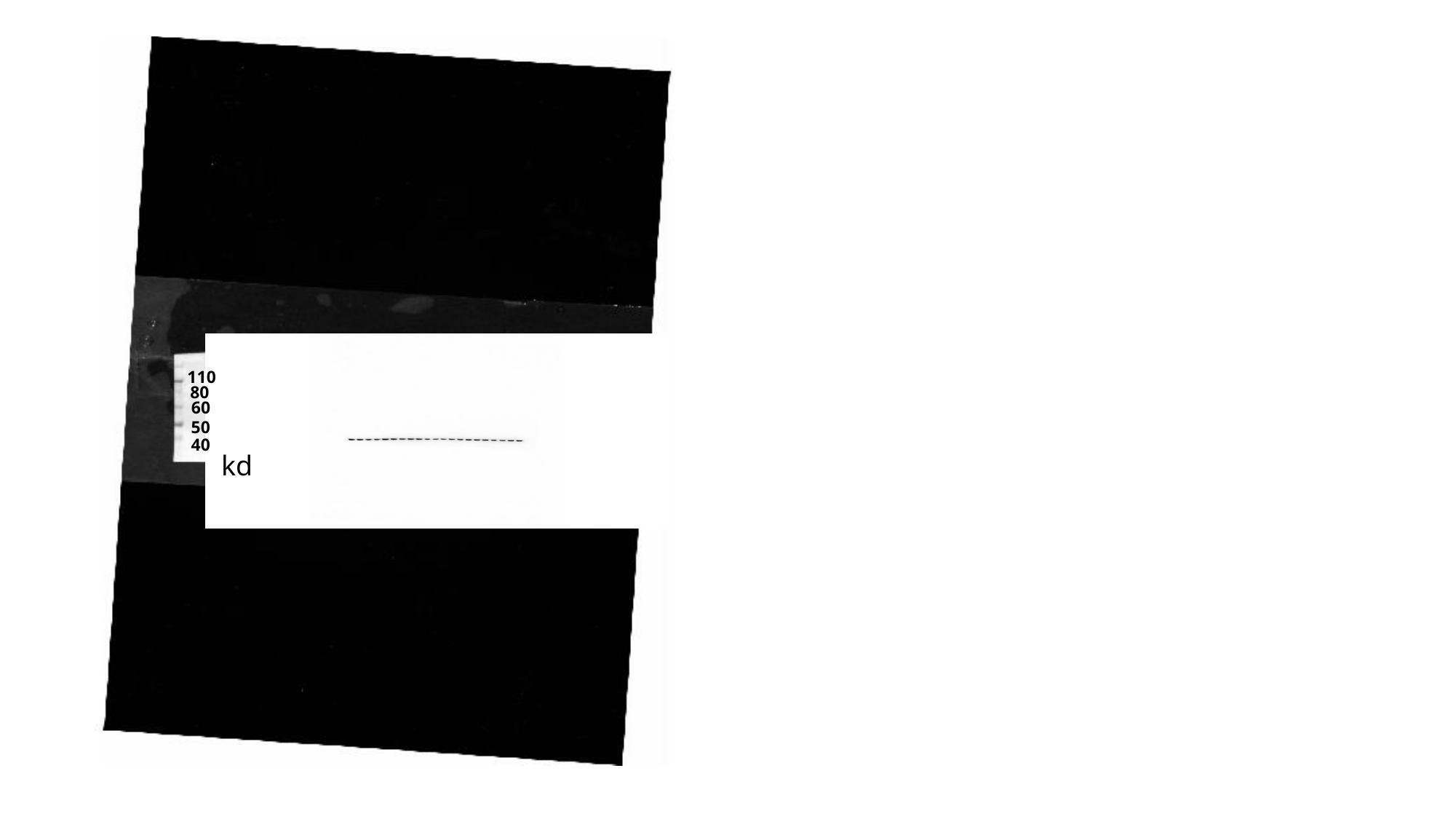

110
80
60
50
40
kd
